# Pharmacological Potential of Flavonoids against Neurotropic Viruses

**DOI:** 10.3390/ph15091149

**Published:** 2022-09-15

**Authors:** Juliana Helena Castro e Silva, Jéssica Teles Souza, Clarissa Schitine, Aníbal de Freitas Santos Júnior, Eduardo Muniz Santana Bastos, Silvia Lima Costa

**Affiliations:** 1 Department of Biochemistry and Biophysics, Health Sciences Institute, Federal University of Bahia, Salvador 40110-100, Brazil; 2 Department of Life Sciences, State University of Bahia, Salvador 41150-000, Brazil

**Keywords:** flavonoid, antiviral, central nervous system infection

## Abstract

Flavonoids are a group of natural compounds that have been described in the literature as having anti-inflammatory, antioxidant, and neuroprotective compounds. Although they are considered versatile molecules, little has been discussed about their antiviral activities for neurotropic viruses. Hence, the present study aimed to investigate the pharmacological potential of flavonoids in the face of viruses that can affect the central nervous system (CNS). We carried out research from 2011 to 2021 using the Pubmed platform. The following were excluded: articles not in the English language, letters to editors, review articles and papers that did not include any experimental or clinical tests, and papers that showed antiviral activities against viruses that do not infect human beings. The inclusion criteria were in silico predictions and preclinical pharmacological studies, in vitro, in vivo and ex vivo, and clinical studies with flavonoids, flavonoid fractions and extracts that were active against neurotropic viruses. The search resulted in 205 articles that were sorted per virus type and discussed, considering the most cited antiviral activities. Our investigation shows the latest relevant data about flavonoids that have presented a wide range of actions against viruses that affect the CNS, mainly influenza, hepatitis C and others, such as the coronavirus, enterovirus, and arbovirus. Considering that these molecules present well-known anti-inflammatory and neuroprotective activities, using flavonoids that have demonstrated both neuroprotective and antiviral effects could be viewed as an alternative for therapy in the course of CNS infections.

## 1. Introduction

The central nervous system (CNS) was long considered a privileged environment due to its cell constitution and the blood-brain barrier (BBB). The BBB is a dynamic interface that limits and regulates the molecular exchanges between the blood and the neuronal tissue or its fluid spaces, counting on the endothelial cells with complex adherents, gap and tight junctions, and astrocytes and pericytes on the basal lamina [1]. Furthermore, the brain parenchyma counts on the resident macrophages named microglia that phagocyte pathogen and present antigens [2].

Despite this, many viruses can infect this system and lead to disease [1,3]. The accumulating evidence shows that the influenza virus, the herpes simplex virus, the enterovirus, the arbovirus, and more recently, the severe acute respiratory syndrome virus 2 (SARS-CoV-2) can invade the brain and, depending on the route of entry, cellular tropism, and mechanism of infection, can trigger a wide range of neuronal symptoms [4,5]. These symptoms include a mild fever to more severe disorders such as encephalitis, encephalopathy, disorientation, loss of memory, and the development of chronic neurological autoimmune conditions [3,6]. Viral infections can also lead to death and impact social life and it is still unclear whether these diseases are caused by a direct cytopathic viral effect or by the inflammatory response against the infected CNS cells [7,8].

Flavonoids are naturally-occurring compounds synthesized from the shikimic acid pathway. They are found ubiquitously in nature and comprise a large group of secondary metabolites formed by a basic three-ring structure with a 15-carbon skeleton comprised of two phenyl rings separated by a heterocyclic ring that is rich in hydroxyl substitutions that are considered as mostly polyphenolic compounds except for the unique group of polymethoxylated flavonoids [9]. These are essential in plant biology, where they act as growth regulators, antibiotics, and antioxidants. Moreover, several biological effects of these molecules on other models of organisms have been described in the literature, which makes them versatile molecules [10] and well-known for their antioxidant, anti-inflammatory, and neuroprotective activities [11]. In this context, flavonoids could be optimal compounds for treating CNS viral infections by acting in a multi-target way, considering that, despite their relatively polar structure, some of them and their metabolites can cross the BBB in different levels of permeability [12,13,14].

In this context, this paper aimed to explore, identify, and summarize the current knowledge of antiviral flavonoids that present activities against neurotropic viruses, in a comprehensive way, including the publications from the last 11 years, and to create a database of promising molecules that are active against viruses that can affect the CNS.

## 2. Methodology

For this investigation, we carried out an exploratory search using the Pubmed database from the National Center for Biotechnology Information (NCBI) of the United States of America (available at https://pubmed.ncbi.nlm.nih.gov/, accessed on 9 September 2022). This platform comprises approximately 30 million citations for MEDLINE’s biomedical literature, life science journals, and online books.

The search was completed in March 2022 using the terms “‘flavonoid’ AND ‘antiviral’” and included the publications dating from January 2011 to December 2021. The Boolean operator “AND” between the terms provides the intercession, restricting the scope of the search in the title, summary, and keywords in order to obtain a more comprehensive and effective outcome for the search result. Four researchers searched through the literature and collected the data. Each researcher sorted through the literature independently during different defined periods of time and screened for the inclusion and exclusion criteria in the titles and abstracts. All researchers revised the included articles. No automatic tools were used in the screening.

The following were excluded: articles not in the English language, letters to editors, review articles and papers that did not include any experimental or clinical tests with flavonoids, and papers that showed antiviral activities against viruses that do not infect human beings. The inclusion criteria were in silico predictions and preclinical pharmacological studies, in vitro, in vivo and ex vivo, and clinical studies with flavonoids, flavonoid fractions, and flavonoid-rich extracts that were active against neurotropic viruses. This selection of results was collectively stored and classified (using Microsoft Excel 2016) according to the year of publication, the type of study, the country of corresponding author, the flavonoid source, the flavonoid name and virus type (Table S1, Supplementary material), which are discussed below.

## 3. Results

Our primary search using the Boolean operators led to 419 results. Upon completion of the screening for the inclusion and exclusion criteria, the investigation resulted in 205 studies that are summarized in Table S1. Among the included articles, 29 dealt with in vivo investigations. However, most of the studies consisted of in vitro studies (155). The in silico predictions were also included in our study and are present in 69 papers.

Regarding the prevalence of the types of viruses studied in the paper, 45 papers studied anti-coronavirus activities, followed by 41 articles that examined anti-influenza activities. Then, 24 studies included anti-herpesvirus activities. arboviruses, including dengue viruses (15), the Zika virus (9), and the Chikungunya virus (1), all of which represent 25 published papers. Lastly, anti-enterovirus activities appeared 13 times in our search.

Several flavonoids appeared more than once and those with more significant data and prevalence are summarized in Table 1.

After selecting the included papers, the full text of the articles was analyzed and the articles were grouped considering the targeted viruses with greater evidence (number of published papers) for the discussion below: influenza viruses, corona viruses, herpes viruses, enteroviruses, and arboviruses.

## 4. Discussion

### 4.1. Anti-Influenza Virus Activities

The influenza virus, a member of the Orthomyxoviridae family, contains ribonucleic acid (RNA) as its nucleic acid and this affects multiple animals. In humans, the influenza strains A, B, and C can infect the respiratory tract and cause seasonal epidemics. Because of their high mutation rate, especially in their membrane glycoprotein neuraminidase and hemagglutinin, it is hard to find a vaccine that is effective [40,41]. Considering this, novel drugs for treating the various types of influenza, especially the subtype A H1N1, seem mandatory.

An influenza infection can present neurological symptoms that include acute encephalitis and febrile seizures. These might be related to other chronic diseases such as the Guillain–Barré syndrome (GBS) [42]. In our search, we observed a first peak regarding the publications on antiviral flavonoids for the year 2015. During this year, five papers examined the different strains of influenza A H1N1 when, during the same year and until mid-2016, there was an influenza pandemic in Europe and Western Asia [43,44].

Among the papers that studied influenza A H1N1, one publication involved an in vitro study and used the bark from stems and leaves in order to obtain methanolic plant extracts with “high flavonoid contents”, measured using the HPLC method and comparing it with the quercetin retention time. In this publication, among a total of five plants, the *Cayratia pedata* (Lour.) Juss. and *Strychnos* spp. stem bark extract had, what the authors called, a “high antiviral activity”. However, none of these was demonstrated to be better than the positive control Oseltamivir. These also had a high concentration of both gallic and coumaric acids [45]. The other publications studied, for example the flavonoids and flavonoid glucosides isolated from the *Matteuccia struthiopteris* (L.) Tod. [46]; baicalin from the *Radix scutellariae* [16]; isoliquiritigenin from the *Glycyrrhiza* spp. [47], and the purchased quercetin, kaempferol, isorhamnetin, diosmetin, and eriodictyol [22], showing the application of flavonoids from different sources. The in vitro studies used different approaches to explore the properties of the flavonoids: an infection using Madin-Darby canine kidney (MDCK) cells [16,22,45,47], an evaluation of their anti-inflammatory effects [47], and the inhibition of the neuraminidase activity [22,46]. Furthermore, the papers that included in vivo analyses used mice infections and the interferon gamma (IFN-ɣ) concentrations [16], the lung infections [16,22], the lung inflammation, and innate immune cells [47] were evaluated. The study performed by Dayem et al. (2015) proposed that hydroxyl and methyl substitution patterns at the carbon 3, 3′ and 4′ positions of the flavonoid skeleton increased the antiviral effect against influenza, as well as with substitutions present using quercetin, kaempferol and isorhamnetin, for example [22].

Interestingly, flavonoids can also present synergistic effects when combined with already approved antiviral drugs. For example, a study described that baicalein can potentialize the antiviral effect of ribavirin, an antiviral drug originally approved to treat hepatitis C, and baicalein activities in vitro in MDCK cells infected with the influenza A H1N1 A/FM1/1/47 strain and treated for 72 h with different concentrations of both molecules [48,49]. It was observed that the optimal synergistic effect was reached when ribavirin (12.5 μg/mL or 25 μg/mL) was combined with baicalein (0.125 μg/mL or 0.25 μg/mL). The interaction also inhibited the expression of the envelope proteins M1 and M2 that had their expression induced in the transfected MDCK cells, in a concentration-dependent manner. The optimal inhibitory effect was obtained using ribavirin 5 µg/mL and baicalein 0.5 µg/mL. In vivo, the authors investigated the effects with ribavirin (50 mg/kg/d) alone or combined with baicalein 100, 200, and 400 mg/kg/d for 24 h in mice. The compounds did not show any toxicity and were effective when combined, thereby reducing the lethality and the body weight loss caused by the influenza infection (A/FM1/1/47 H1N1 strain), with better results using the synergic treatment with ribavirin (50 mg/kg/d) and baicalein 400 mg/kg/d, which was 100% effective when compared to the untreated control [48].

Baicalin, the baicalein aglycone, was also studied against influenza A H1N1 in both in vitro and in vivo models, as well as influenza A H3N2. The authors created a viral replication in the A519 lung adenocarcinoma cell line treated with the flavonoid for 24, 48, and 72 h. In the 24 h treatment, the flavonoid was effective and reduced the virus titer at the three timepoints against the H1N1 (EC_50_ = 17.04 µg/mL) and H3N2 (EC_50_ = 19.31 µg/mL) strains. The in vitro treatment also reduced the expression of proteins associated with the viral replication of M1 and MP and upregulated the expression of miRNA miR-146a, which was associated with the increased viral replication in some cases due to the impairment of the interferon (IFN) response, and then decreased in the infected cell line. It was shown in vivo as well, that baicalin (dose not mentioned) improved the lung condition at 6 days post-infection, which increased the survival rate and acted via the downregulation of miR-146a and the upregulation of interferon-alpha (IFNα) and beta (IFNβ) in the bronchoalveolar fluids [17].

Nayak et al. (2014) [15] used in vitro, in vivo, and in silico techniques to show the baicalin effects against the influenza H1N1 pdm09 strain. They tested the baicalin (0.5–320 µM) toxicity for 4 days on infected A519 cells and evaluated the cell survival using the MTT assay and the viral inhibition for 72 h using neural red. Baicalin did not appear to be toxic and reduced the viral infection in vitro (IC_50_ ~ 18 µM). In vivo, baicalin (10–220 mg/kg/d, twice a day for three days) was tested in infected mice and it was shown that baicalin reduced the virus titer in lung homogenates and reduced the nucleocapsid protein expression for up to 24 h after infection. Furthermore, the level of important cytokines for a viral response was analyzed by the quantitative reverse transcription polymerase chain reaction real time (qRT-PCR). IFNα and β, the interferon regulatory factor 3 (IRF-3), oligoadenylate synthetase 1 (OAS1), and RNase-L were increased compared to the control group. In both in vitro and in vivo experiments, baicalin decreased the expression of the viral NS1 protein, a very conserved protein, analyzed by Western blotting. Furthermore, the authors investigated the in silico activities of baicalin in docking NS1, which suggested that baicalin interacted with the receptor binding domain (RBD) of NS1. This activity is also suggested in an inhibition fluorometric in vitro assay with baicalin and the NS1 protein and the NS1-RBD [15].

The neuroprotective flavonoid rutin and its aglycone quercetin were tested against influenza in in vitro and in silico models. Kashiwada et al. (2012) [50] used the *Moringa oleifera* Lam. leaves in order to extract five flavonoid glucosides: three quercetin derivatives (isoquercitrin, quercetin 3-O-β-D-(6″-O-malonyl)-glucoside, and quercetin 3-O-β-D-(6″-O-3-hydroxy-3-methylglutaryl)-glucoside) and two kaempferol derivatives (astragalin and kaempferol 3-O- β -D-(6″-O-malonyl)-glucoside). The paper focused much more on the isolation and chemistry of these compounds; however, it was mentioned that, among the tested flavonoids, the 3-O-β-D-(6″-O-malonyl)-glucoside inhibited the neuraminidase from influenza A H1N1 in a colorimetric assay with moderate activity (IC_50_ 25.3 µg/mL) [50].

In a recent paper, Ling et al. (2020) [35] studied rutin and its quercetin derivatives, quercitrin and isoquercitrin, and a flavonoid extract from the *Houttuynia cordata* Thunb. (HCF), which is used in folk medicine for treating pneumonia. In mice, HCF (50, 100 and 200 mg/kg) reduced the viral-induced weight loss and ameliorated the lung condition. The extract also induced a decrease in the monocyte chemotactic protein 1 (MCP-1), interleukin-8 (IL-8), and the tumor necrosis factor-α (TNF-α) levels in the lung tissue that was dosed using ELISA. The extract also reduced the expression levels of the Toll-like receptors TLR3/4/7 and the transcription factor nuclear kappa B (NF-κB). This reduction might be a strategy in regulating the chronic immune response against viruses. In vitro, the treatments with rutin and quercitrin (100 µg/mL) reduced the viral infection in the MDCK cells. The results suggested that the antiviral activity might be associated with the neuraminidase inhibition since rutin, quercitrin, and another flavonoid called hyperin, inhibited the neuraminidase activities in vitro. Moreover, the authors investigated the immune response of the flavonoids in the supernatant of the infected cells treated with different inflammatory immunostimulants. HCF (50, 100 and 200 mg/kg), rutin, quercitrin, and hyperin (100 µg/mL) reduced the levels of the cytokines IFN-β and IL-6 in the supernatant of cells treated with polyinosinic:polycytidylic acid or imiquimod and reduced the IL-6 supernatant levels of cells treated with lipopolysaccharide (LPS) [35].

The neuraminidase inhibition by quercetin is shown in silico, together with other flavonoids (catechin, narigenin, luteolin, dinatin, vitexin, chrysin, and kaempferol). Quercetin, chrysin, dinatin, and kaempferol were considered the most promising flavonoids that presented the lowest AutoDock energies (−6.8), compared with the oseltamivir used in influenza A therapy (−5.8) [23].

Another flavonoid studied for its anti-influenza activity is lutein. Influenza viruses contain a single-stranded, RNA-dependent RNA polymerase (RdRp), which is necessary for the virus in order to synthesize its own 5′-mRNA cap. This RNA polymerase PA subunit leads the cleavage of the RNA segment and, currently, there is one FDA-approved drug that targets this subunit: Baloxavirmarboxil. Reiberger and colleagues developed a screening assay and determined the inhibitory potency of 38 flavonoids in inhibiting PA, of which luteolin (IC_50_ of 73 ± 3 nM) and its 8-C-glucoside orientin (IC_50_ of 42 ± 2 nM) were the most potent inhibitors [51].

The most abundant catechin of green tea (*Camellia sinensis* (L.) Kuntze), epigallocatechin-3-gallate (EGCG), has been shown to bear an antiviral activity against a wide range of viruses through diverse mechanisms, especially influenza viruses, by exerting the irreversible virucidal effect [52]. Using an innovative approach, Cheong and colleagues showed that EGCG, besides exerting the virucidal effect, can serve as a novel vaccine adjuvant for the influenza vaccine, using recombinant and influenza-derived hemagglutinin (HA) as the antigen vaccine in a mouse model. The data indicate that green tea-derived EGCG induces a more robust increase in antibodies after the HA sensibilization than does the alum, a classic vaccine adjuvant. In addition, the combination of EGCG and alum induced higher levels of the neutralizing antibodies, thereby demonstrating a synergistic effect of EGCG and Alum [53].

A study by Li et al., 2021 [54], characterized four honeysuckle extracts against influenza. The acidic flavonoid mixture showed the most effective antiviral activity against H1N1 and H3N2 by inhibiting the viral replication with EC_50_ values of 3.8 μg/mL and 4.1 μg/mL, respectively, in the MDCK-infected cells. The inhibitory efficacy of the four honeysuckle extracts on the influenza A virus in vivo was also evaluated in an H1N1-infected mouse model administered intranasally with a lethal dose of the H1N1 virus. At 2 h post-infection, the drugs were delivered orally to mice once daily for 5 days. Treatment of the mice with 600 mg/kg/d of the honeysuckle acids extract, the flavonoid extract, the total extract, or the acid-flavonoid mixture increased survival to 30%, 10%, 10%, and 20%, respectively. Moreover, in order to investigate the antiviral mechanisms, the study indicated that among the four honeysuckle extract treatments, the flavonoid extract showed the best anti-neuraminidase activity of the influenza A virus, cleaving the sialic acid residues of the viral receptor required for releasing the progeny viruses [54].

Chrysin has a wide range of pharmacological and biological activities, including anti-inflammatory, anti-cancer, antibiotic, and antifungal properties. Kim and colleagues in 2021 demonstrated that chrysin had antiviral activities against influenza by inhibiting the autophagy, a process that helps cells to degrade the pathogens in the innate immune response and leads to the viral clearance, but it is also a process that is replicated and used by some viruses, such as influenza A. Chrysin increased the cell viability, reduced the hemagglutinin and blocked the autophagy (shown by the reduction of the LC3 autophagosome and the increase in the mTOR protein levels) in infected A549 cells treated with 50 µM chrysin for 24 h [39].

These findings together suggest that flavonoids can act as antiviral drugs for influenza, especially influenza A H1N1 by inhibiting the important virus proteins for cell infection, while they can modulate the inflammatory responses that are helpful against the infection and the chronic progression of the disease.

### 4.2. Anti-Coronaviruses Activities

Recently, the COVID-19 disease caused by the severe acute respiratory syndrome coronavirus 2 (SARS-CoV-2) has become a pandemic. It has been described that the virus also infects the CNS, which can generate neurological symptoms and contribute to the impairment of the respiratory capacity. The body of data focusing on how the infection can chronically affect COVID-19 patients or how the virus invades the CNS is increasing, but there are still many gaps to fill in this field. Some studies suggest that this might occur via blood or via the olfactory nerve and/or trigeminal ascendant pathway [55,56]. Moreover, in 2020, a case of a SARS-CoV-2 infection was reported that could be related to the acute induction of Parkinsonism [57,58].

Nowadays, a SARS-CoV-2 infection is recognizably related to the acute and persistent neurological and psychiatric symptoms that affect up to 1/3 of the infected patients, who do or do not require hospitalization. These can range from anosmia, a feeling of “brain fog”, attention impairment, to depression and anxiety and other severe neurological events such as stroke [59,60,61].

In total, our search has identified 78 publications that mention some activity against coronaviruses. In one of these papers, the authors evaluated the anti-SARS-CoV activity of synthetic 7-O-Arylmethylquercetin derivatives using an in vitro spectroscopic assay of NTPase/helicase coronavirus inhibition, proteins that are involved in different stages of the virus life cycle. The most promising compound (2R, 7-(4-Chlorobenzyloxy)-2-(3,4-dihydroxyphenyl)-3,5-dihydroxy-4H-chromen-4-one) had a Cl substitution on Carbon 4″ and presented (IC_50_ 20.9 µM for NTPase and 4.1 µM for helicase) [62]. Another study tested the activity of afzelin, a flavonoid glycoside from the *Euphorbia neriifolia* (L.)*,* against the human fibroblasts cell line (MRC-5 cells) that was infected with HCoV in the XTT viability assay. The authors mentioned this activity but did not show any quantitative data [63].

In another study, the authors tested 64 flavonoids in an in vitro model of the inhibition of the synthetic Middle East respiratory syndrome-coronavirus (MERS-CoV) protein nsp5. Using a fluorimetric assay, they found that herbacetin, isobavachalcone, quercetin 3-β-d-glucoside, and helichrysetin inhibited nsp5 and had the best results among the analyzed flavonoids, with IC_50_ values of 40.59, 35.85, 37.03, and 67.04 μM, respectively. They concluded that the chalcone skeleton flexibility might be important for the activity because the helichrysetin and isobavachalcone activity was better than the others [64].

More recently, another study, using the same methods as Jo et al. (2019), investigated whether flavonoids could inhibit the SARS-CoV 3CL protease, which is essential for releasing viral non-structural proteins, using both a fluorogenic (FRET) and tryptophan fluorescence-based in vitro inhibition assays. They found that herbacetin, rhoifolin, and pectolinarin had the best results and were capable of inhibiting the 3CL protease with IC_50_ values of 33.17, 27.45, and 37.78 μM, respectively [44]. In another paper published in 2020, the authors also investigated the flavonoid potential of inhibiting the SARS-CoV-2 3CL protease. By applying a library of 70 flavonoids and using the same in vitro strategies as the former, the authors described that baicalin, herbacetin, and pectolinarin presented the best inhibitory activities with IC_50_ values of 34.71 µM, 53.90 µM, and 51.64 µM, respectively [65].

Basu and colleagues studied the in silico activity of the flavonoids hesperidin and chrysin in the binding to the SARS-CoV-2 spike protein binding site and to the angiotensin-converting enzyme 2 (ACE2), thereby allowing cell entry. In this study, anthraquinone, rhein, chloroquine, and hydroxychloroquine bindings were also evaluated. However, the authors found that chrysin was shown as the most competent inhibitor of the spike protein-ACE2 binding, considering the number of interactions with both the spike protein and ACE2 [66].

Another study by Song and colleagues and published in 2021 showed that baicalein, an active compound isolated from the *Scutellaria baicalensis* Georgi (Huangqin), has therapeutic effects against SARS-CoV-2, in in vivo and in vitro models. Their data showed that a baicalein pre-treatment at the concentration range of 0.1–0.3 μM could completely protect the Vero E6 cultures against SARS-CoV-2. In addition, baicalein inhibited the loss of body weight, reduced virus load, relieved lung injury in mice infected with SARS-CoV-2, and alleviated the LPS-induced acute lung function injury in mice when orally administrated at a dose of 50 mg/kg [67]. In a separate study, the data demonstrated that the ethanol extract from the *S. baicalensis* Georgi (Huangqin) and its major component, baicalein, inhibited the SARS-CoV-2 3CLpro activity in vitro with a similar range of IC50, of 8.52 µg/mL and 0.39 µM [68].

Epigallocatechin-3-gallate (EGCG) has antiviral activities against a variety of viruses, as we mentioned previously, by directly inhibiting the viral binding to the host cell. EGCG was shown to block the SARS-CoV-2 cell entry by the reduction of the luciferase activity in the HEK293T-ACE2 cells transduced with the SARS-CoV and SARS-CoV-2 spike pseudotyped-protein lentiviral vector. The 24 h incubation with the EGCG induced dose-dependent inhibited the cell entry with IC_50_ values of 4.28 µg/mL for SARS-CoV and 2.47 for SARS-CoV-2. Epicatechin (EC) was also tested in the study but showed that IC_50_ was higher than 20 µg/mL. ECGC also inhibited a viral replication with the pseudotyped lentiviral vector and the infection virus using the virus plaque reduction assay [30].

The SARS-CoV enveloped protein E is highly conserved between SARS-CoV and SARS-CoV-2, which participates in viral packaging and reproduction, also interacts with other proteins and self-assembles in order to create ion channels [69]. Breitinger and colleagues [70] induced the SAS-CoV E protein expression in HEK293 cells and analyzed the E protein inhibition, the channel activity using patch-clamp electrophysiology and a cell viability assay after treating these cells with 10 flavonoids: kaempferol, quercetin, naringenin, apigenin, nobiletin, genistein, EGCG, resveratrol, 6-gingerol, and 8-gingerol. Their data indicate that epigallocatechin and quercetin were the most effective, with IC_50_ values of 1.5 ± 0.1 and 3.7 ± 0.2 nM, respectively, and that are similar to the potency of rimantadine (IC_50_ = 1.7 ± 0.6 nM), which is a well-known antiviral drug, thereby suggesting that these compounds could contribute to the development of novel antiviral drugs that suppress the virus activity and proliferation [70].

### 4.3. Anti-Herpes Virus Activities

Herpes simplex viruses (HSV-1 and HSV-2) are DNA viruses of the Herpesviridae family. HSV infections usually lead to recurrent symptoms such as facial, lips and oral cavity, and genital ulcerations [71,72]. In the CNS, HSV-1 and HSV-2 are related to encephalitis, meningitis, blindness, and even death [73]. Moreover, after the primary infection at the mucosal site, the virus establishes a latency in the trigeminal ganglion, where it remains throughout the life of the host. For this reason, its prevalence increases with age and asymptomatic hosts remain contagious. Therefore, HSV-1 can reactivate at any time, and there are no prevention vaccines or treatments [74].

The treatment of HSV infections requires the continued use of synthetic drugs such as acyclovir (ACV) and its analogue, penciclovir (PCV) [75]. However, this long-term use can lead to resistant HSV strains, which makes the search for novel drugs imperative. Among the papers analyzed, we found different active flavonoids against the HSV strains. In one study, authors tested the aqueous extract from the plant *Houttuynia cordata* Thunb. from the Saururaceae family, used in Chinese folk medicine. The dried leaf extract also contains flavonoids such as quercetin, rutin, and houttuynoid A (HA). The aqueous extract (pretreatment, EC_50_ (0.692 mg/mL) showed a positive effect against herpes strains and reduced the viral cytotoxicity in an in vitro model of the infection in the Vero cell line. The authors concluded that the extract inhibited the expression of a viral glycoprotein D (gD), which can interfere in the viral fusion and suppress the viral entry and replication (0.0005 to 0.1 mg/mL). The extract and isolated flavonoids quercetin, rutin, and isoquercetin attenuated the pro-inflammatory molecule NF-ĸB expression, especially at the beginning of the infection [24].

Another study also explored the effects of the *Houttuynia cordata* Thunb. aqueous extract NF-κB inhibition. In this study, the extract (45, 150, and 450 µg/mL) inhibited the HSV activity in vitro in 100% and suppressed the NF-κB virus-induced expression. Other flavonoids previously described as present in the extract also inhibited the viral activity, such as quercetin, quercitrin, and isoquercitrin (10 µM) [76].

Other authors investigated the *H. cordata* Thunb. water extracts and compounds against the HSV. Li and colleagues (2017) [77] tested the *H. cordata* Thunb. and houttuynoid A (HA) against the HSV (HSV-1) using in vitro and in vivo assays. The authors analyzed the antiviral activity of the compounds in the in vitro methods, also using the Vero cell line. The authors described that the *H. cordata* Thunb. extract and HA reduce the β-galactosidase activity (IC_50_ = 107.10 ± 11.48 mg/mL and 23.50 ± 1.82 mM, respectively) and reduced the viral fusion and cell plaque formation. This manuscript also described the HA (100 µM) treatment for up to 8 days post HSV infection in mice through broken skin. The result showed that HA improved the lesions and viral loads in skin tissue [77].

Many other flavonoids are described as anti-HSV. For example, the study by Fukuchi et al. (2016) [78] analyzed the aqueous extract from the roots of the licorice tree *Glycyrrhiza glabra* (L.) (Fabaceae family) and different partitions. However, only its flavonoid-rich fraction presented a reasonable in vitro anti-HSV activity and recovered cell viability (EC_50_ = 13 µg/mL) [78].

Li et al. (2020) [34] demonstrated that the flavonoid myricetin (1.25–20 µM) significantly reduced the number of plaques in the HSV-1- and HSV-2-infected Vero cells, suggesting the direct inactivation of the viral particles. Myricetin also reduced the viral cytotoxicity at lower concentrations than acyclovir. It has been also shown that myricetin can block an HSV infection via gD and negatively regulate the epidermal growth factor receptor (EGFR), phosphatidylinositol-3-kinase (PI3K), and the Protein kinase B (PKB) cell signaling pathway, which can reduce infection and replication of the HSV. In vivo, myricetin (2.5 and 5 mg/kg, intraperitoneal) increased the rat survival rate and body weight in rats and reduced the virus titers in the lung when compared with the placebo and with the animals treated with acyclovir (10 mg/mL). From this perspective, these pieces of evidence suggest that flavonoids can act as anti-HSV in a multi target way (e.g., the inhibition of HSV proteins and cell signaling pathways or reducing cell cytotoxicity and viral replication) [34].

Additionally, the viral infection-mediated tissue damage that is associated with oxidative stress plays an important role in their pathogenesis [79]. An HSV-1 infection induces the excessive production of the reactive oxygen species (ROS), which can be beneficial for eliminating invading pathogens. However, it can also increase the virus replication in infected cells, inhibit cell proliferation and induce cell death. Therefore, the inhibition of the HSV-1-induced ROS by flavonoids could be a so-far non-exploited additional mechanism of action of flavonoids against HSV-1 [79].

### 4.4. Anti-Enteroviruses Activities

Enteroviruses are a genus of single-stranded RNA of the *Picornaviridae* family. The enteroviruses can cause several respiratory and neurological disorders in humans and in animals [80,81]. Enterovirus 71 (EV71) and coxsackievirus are the most frequent enterovirus species that cause infections of the extremities (feet, hands, and mouth syndrome), severe neurological infections in children and babies (HFMD), aseptic meningitis, as well as encephalitis and acute flaccid paralysis (AFP, polio and polio-like syndromes) that can even lead to death [82,83].

For the EV71, the complicated illness is rare but especially concerning when the patient is an infant, with possible sequelae development up to 7 years after the primary symptoms due to lesions on the brain stem or spinal cord [84,85]. Authors claim that the lesions in the EV71-induced AFP are the consequence of inflammation-induced neuronal death, which, currently, relies on no effective therapeutic strategies available [86].

Wang et al. (2014) [87] used in silico approaches (Autodock 4.0) to predict whether flavonoids could bind to 3C^pro^ Enterovirus 71 protease, a protein involved in RNA binding and replication. Crysin (7-dihydroxyfavone, CR) and its phosphate ester diisopropyl chrysin-7-yl phosphate (CPI) were both able to interact with 3C^pro^ at various amino acid sites (LEU4, LEU8, SER111, MET112, PHE113, and PRO115) with a negative binding energy (−5.55 kcal/mol). This study also showed that the flavonoids inhibited the EV71 plaque formation and the 3C^pro^ activity in vitro as well, when compared to controls: 40% for the inhibition of CR and 60% for the inhibition of CPI. A similar study showed that 7-hydroxyflavone (HF) and its phosphate ester (FIP) could also bind to 3C^pro^ and strongly inhibited the viral activity in cells [87].

In a study by Li et al. (2015) [19], the flavonoid baicalin (6.25, 12.5, 25, and 50 μg/mL) showed an inhibitory activity against an EV71 infection in a concentration-dependent manner. Furthermore, baicalin (50 μg/mL) treated for 4 or 8 h reduced the viral RNA titers. This inhibition seems to be independent of the direct viricidal or prophylactic effect and inhibitory viral absorption. It showed that baicalin (50 μg/mL) decreased the expression of the FasL protein, a member of the tumor necrosis factor (TNF) family, and caspase-3, a marker of apoptosis, in an in vitro model of the infection in human embryonic rhabdomyosarcoma (RD) cells [19].

The flavonoid apigenin was identified as the active compound of the *Paulownia tomentosa* Steud. flower extracts against the EV71 infection in vitro. In a study by Ji et al. (2015) [36], apigenin inhibited the viral activity (EC_50_ = 11.0 µM) and seemed to target the EV71 ribosome interaction with the nuclear ribonucleoproteins (hnRNPs) that cause the viral translation [36].

In our search, only five studies mentioned anti-coxsackievirus activities. Among these, interestingly, was a study by Galochkina et al. (2016) [88] that showed that a quercetin derivative can act as an antiviral and anti-inflammatory and improve the infection condition. The flavonoid dihydroquercetin/taxifolin (100 µg/mL) was isolated from larch heartwood (*Larix sibirica* Ledeb.) and the authors tested their effects in both in vitro and in vivo approaches. The authors described that the dihydroquercetin (75 or 150 mg/kg once a day for five days) decreased the coxsackievirus B4 (CVB4) virus titers in the pancreatic tissue in mice, normalized the antioxidant levels of the pancreatic tissue, and reduced the inflammatory infiltrate in mice lungs when compared with the placebo [88].

Considering that an EV71 infection is dangerous for infants, Dai et al. (2019) [37] showed the efficacy of the intraperitoneal administration of eight flavonoids against the lethal intracranial EV71 infection in newborn mice (P0). Following the calculation for the optimal EC_50_ in an in vitro infection model of HEK cells, the authors used flavonoids for 7 days and showed that all of the flavonoids increased the survival rate of the pups at some level, especially isorhamnetin at 10 mg/kg/day, which increased the survival rate to 100% despite having a relatively high EC_50_ of 60.7 µM for the antiviral activity in vitro, followed by 10 mg/kg/day of luteolin (91.67%, EC_50_ 13.5 µM) and 50 g/kg/day of apigenin (88.89%) [37].

### 4.5. Anti-Arbovirus Activities

Arboviruses (arthropod-borne virus) comprise a diverse group of families of single-stranded RNA viruses that depend on a wide variety of arthropod vectors for the transmission and infection of susceptible hosts [89]. Although they are zoonotic pathogens, some arboviruses can infect humans and, in some cases, lead to severe illnesses. These conditions have been associated with severe neurological complications such as meningitis, encephalitis, encephalomyeloradiculitis, congenital microcephaly, lysencephaly, hydrocephalus, parenchymal calcifications, seizures, and GBS [90,91,92,93].

Most arboviruses that cause human diseases belong to the Bunyaviridae, Reoviridae, Flaviviridae, and Togaviridae families. The last two classes were the most cited in the articles included in this study [26,31,33], especially: the four serotypes of the dengue virus (DENV1, DENV2, DENV3 and DENV4), the Zika virus (ZIKV), (these two members of the family Flaviviridae (genus Flavivirus)) and Chikungunya virus (CHIKV), (member of the Togaviridae (genus Alphavirus) family)The particular interest in the discovery of the phenolic compounds with the antiviral action for these viruses is mainly due to the imminent risk of epidemics, the absence of effective vaccines, and their potential neurological complications which are causes of hospitalization and death by arboviruses [89,91,94,95].

The knowledge of viral biology led to the discovery of potential pharmacological targets associated with the control of the arbovirus replication, such as the non-structural proteins (NS1, NS2a, NS2b, NS3, NS4a, NS4b, and NS5) that modulate the processes of the viral replication, assembly, proteolysis, and regulation of the cellular immune response [96,97]. For the ZIKV, the articles investigated the NS2B-NS3 protease inhibitors. For the DENV, in addition to the NS2B-NS3 protease, the authors investigated the inhibitors for the multifunctional NS3 enzyme. The CHIKV genome encodes four non-structural proteins (nsP1, nsP2, nsP3, and nsP4), that are associated with the viral mRNA transcription and translation process, in addition to other structural proteins [98]. The cysteine-protease domains nsP1, nsP2, and nsP3 were cited as an important target for candidates for the alphavirus protease inhibitors in the articles investigated [99].

#### 4.5.1. Anti-Dengue Virus (DENV) Activities

Although a DENV infection is not considered as neurotropic, neurological manifestations of it are present in 6 to 20% of the cases. The antibodies from the IgM class and DENV RNA can be found in the cerebrospinal fluid (CSF) of infected patients, which suggests the BBB invasion and parenchyma infection [100].

The first report found in the literature on the antiviral potential of the phenolic compounds against the DENV dates from 2006. A study showed the inhibitory activity of flavonoid derivatives present in the root of the *Boesenbergia rotunda* (L.) Mansf., against the NS3 protease of the DENV2 in an in vitro model. In the last 11 years, a series of flavonoids have been tested for the different serotypes of the DENV [101]. Moghaddam et al. (2014) [18] demonstrated that baicalin (50 μg/mL), the main metabolite of baicalein, had an effect against the DENV2 replication (IC_50_ = 13.50 ± 0.08 μg/mL). In addition, the flavonoid induced the inactivation of the free viral particles and inhibited the viral adsorption stage in the host cells [18].

In a study by Frabasile et al. (2017) [102], naringenin was tested against the DENV in an in vitro assay using Huh7.5 cells and peripheral blood mononuclear cells (PBMCs). The results showed the flavonoid ability to inhibit the viral replication in Huh7.5 cells at a concentration of 250 μM for up to 24 h after exposure to the four different serological types of viruses. In the model using PBMCs, a similar result was observed for cells infected with the DENV4 and treated with 62.5 μM naringenin [102]. In another experimental in vitro study, the authors explored the activity of the aqueous extract from the roots of the Chinese medicinal herb *Scutellaria baicalensis* Georgi. The extract inhibited the different stages of the viral replication and the reduced infection by the four serotypes of the DENV (IC_50_ values ranging from 269.9–369.8 μg/mL) [103].

More recently, Care et al. (2020) [33] tested the kaempferol (100 μM) antiviral activity against the DENV2 and the Japanese encephalitis virus (JEV) infection, another important flavivirus associated with neurological complications. The in vitro treatment with kaempferol on BHK-21 cells induced a decrease in the viral production and inhibition of the JEV infection. Surprisingly, for the DENV, there was an increase in the viral infection, associated with the increased expression of the viral E protein and host cell protein GRP78, which suggests the endoplasmic reticulum stress by a mechanism not yet understood [33]. In silico analyses have helped to understand the relationship between the chemical structures of flavonoids and their ability to inhibit the dengue virus polymerase [32]. Using this same approach, several flavonoids, including quercetin and myricetin, were shown to be able to inhibit the viral activity related to the NS2B-NS3 protease complex [25]. Likewise, the DENV2’s NS2B-NS3 protease complex can be inhibited mainly by quercetin [104].

The only in vivo study using flavonoids in the presence of the DENV infection in our search was limited to evaluate the renal and liver function in mice that had intravenous infusions of two synthetically modified flavonoids, 6,8-dibromopinostrobin and 6,8-dibromopinocembrin, based on the pinostrobin skeleton. The analysis showed that the flavonoids at the 10 mg/kg single dose did not show any significant toxicity to these animals up to 8 days after administration [105].

#### 4.5.2. Anti-Zika Virus (ZIKV) Activities

The fast geographical expansion of the ZIKV transmission caused outbreaks in Brazil with associated cases of congenital microcephaly in newborns in 2016, a fact that has raised interest in new antiviral therapies [106]. The mechanism of the congenital microcephaly induction is still under debate, but studies suggest that the ZIKV crosses the placental barrier and BBB after infecting both the blastocysts and the brain endothelium [107] and, later the neuronal stem cells (NSCs), by inducing an early cell death via the endoplasmic reticulum stress, apoptosis and through the inhibition of the Akt-mTOR viability pathway by NS4B [108,109].

In our research, Carneiro and colleagues were the pioneers in the investigation of the antiviral effects of phenolic compounds against the ZIKV, in an in vitro model of the infection in the Vero E6 cells. The results showed that the flavonoid EGCG inhibited viral entry into the cells (90%) at higher concentrations (>100 μM). The authors suggest that the mechanism of inhibition is related to the ability of the polyphenol to destroy the phospholipids in the viral envelope [28].

Later, a study evaluated the inhibitory potential effect of 22 polyphenolic compounds, including myricetin, astragalin, rutin, EGCG, epicatechin gallate (ECG), galocatechin gallate (GCG), and luteolin (100 μM) against the ZIKV infectivity. Their results demonstrated an important structure-activity relationship in inhibiting the ZIKV NS2B-NS3Pro with flavonoids IC_50_ ranging from 22 ± 0.2 μM to 112 ± 5.5 μM, the most powerful ones having 3 (-OH) substitutions in the C3′, C4′, and C51 from the B ring [29].

More recently, the antiviral activity of flavonoids was described via a mechanism involving the inhibition of the ZIKV NS2B-NS3 protease in the Vero cells. The treatment with the flavonoids galangin, kaempferide, quercetin, myricetin, and EGCG (IC_50_ from 0.02 to 14.36 μM) inhibited this protease and showed that these values decreased when the number of hydroxyl groups of the compounds increased, which showed a clear structure-activity relationship [26]. Cataneo et al. (2019) [110] demonstrated that the flavonoid naringenin inhibited four different ZIKV strains in an experimental model of the infection in human A549 cells, for up to 24 h after the establishment of the infection with IC_50_ of 58.79. In addition, the molecular docking assay showed that naringenin interacts with the ZIKV protease domain NS2B-NS3 [110].

#### 4.5.3. Anti-Chikungunya Virus (CHIKV) Activities

In 2018, a systematic review including 94 publications and a total of 856 patients showed that the neurological complications represent the most frequent severe complication by the CHIKV infection, and encephalopathy as the most prevalent one (40.5%). Furthermore, the chronic peripheral comorbidities, most commonly GBS, were described in 72 patients. When the treatment was mentioned for these patients, it was mostly via immunoglobulin administration, which still represents an expensive form of therapy, especially in endemic environments [91].

The first study that mentioned the antiviral effect of flavonoids against the CHIKV demonstrated that the phenolic compounds with a 5,7-dihydroxyfavone structure (more specifically apigenin, chrysin, naringenin, and silybin) inhibited the CHIKV infection in a stable replicating cell line [38]. Later, Weber et al. (2015) [27] demonstrated the anti-CHIKV activity of the EGCG inhibition of the in vitro infection in HEK 293T cells, by blocking the binding and entry of the lentiviral vectors that expressed the CHIKV envelope proteins in the target cells (IC_50_ 6.54 μg/mL) [27].

The flavonoid baicalin was described as an anti-CHIKV by Oo (2018) [20]. The in vitro study evidenced that the flavonoid had the potential to inhibit the viral action using different cell lines with EC_50_ ≈ 7 µM. It was suggested that baicalin acts directly on the virus envelope and/or glycoproteins and inhibits mainly the early stages of the viral replication cycle. In addition, a dose-dependent reduction was observed in the expression of the CHIKV E2, nsP1 and nsP3 proteins, which are involved in the binding of the virus to the target receptors of the host cells, in the process of virus RNA synthesis and regulation of the cell metabolism [20].

The in silico screening performed by Keramagi and Skariyachan (2018) [31] pointed out to a series of natural compounds present in medicinal plants, including kaempferol, chymopain, and gossypetin. The study described that the flavonoids had a good binding potential against the CHIKV and DENV targets. In the case of the CHIKV, the main targets were the non-structural proteins NSP2 and NSP3, involved in the viral RNA synthesis and the inhibition of cellular proteins, and the E2 envelope protein, involved in the binding to the host cell receptor [31].

## 5. Pharmacokinetic Properties of the Flavonoids and the BBB

The BBB has a crucial role in allowing nutrients and non-nutrients (such as (poly) phenols) to the brain parenchyma and the physicochemical characteristics of polyphenols, such as solubility, molecular weight, lipophilicity, and pKa, are of great relevance in their bioavailability for the CNS [111]. Despite their structural variability, the accumulating evidence shows that flavonoids can cross the BBB [12,13,14].

Youdim and colleagues, in 2004, compiled in vitro and in situ studies in order to analyze the permeability of flavonoids through the BBB. They showed that flavonoids were found in all brain regions (cerebellum, cortex, hippocampus, hypothalamus, striatum, superior colliculus, and medulla) during the in vitro tests, indicating that flavonoids with different structures can reach different regions of the brain [12].

Another study indicated that the conjugated methylated metabolites of the flavonoids in the small intestine and liver have a greater BBB permeability [13]. A more recent study has shown for the first time that polyphenol metabolites are also able to cross the BBB endothelium at physiologically and relevant concentrations [14]. However, some authors indicate that the expression of the specific ATP-dependent carriers, such as the glycoprotein P expressed at the BBB, could also mediate the polyphenol transport [112].

As mentioned earlier, the chemical structure of the phenolic compounds alters the efficiency in the ability to cross the blood-brain barrier. In addition, it defines whether they are absorbed in the small intestine or reach the colon in order to undergo a microbial catabolism, which can also be the process responsible for the various biological effects verified in the studies using an oral flavonoid administration. The metabolic transformations that occur in the intestine or liver generally make molecules more hydrophilic and therefore are less likely to reach the CNS. Unabsorbed compounds and phenolic metabolites released into the intestine via the enterohepatic recirculation reach the colon where they are catabolized by gut-resident microbes. The changes induced by the microbiota, especially related to the action of the specific enzymes of the intestinal bacteria, convert these metabolites into less complex molecules that can reach up to 95% of the initial ingested polyphenol concentration [113]. Therefore, the gut microbiota is also an essential component of the biological activities and bioavailability of the polyphenols, even without crossing the BBB [114].

## 6. Limitations of the Present Study

Our study also presents limitations regarding the time frame selected for this study, which comprises the last 11 years. Although Pubmed comprises more than 33 million published scientific papers, the use of more than a single repository as the source of articles would be ideal.

Furthermore, in all types of studies, many of the viruses discussed in this review lack some antiviral/neuroprotective standard treatment to be used as the positive control, which makes the comparison more difficult and explicit for the urge for antiviral therapies.

Also, for some clinically important neurotropic viruses such as the those from the Paramyxoviridae family, which includes the respiratory syncytial virus, the measles virus, and the mumps virus, there were very few studies that indicated the activity of flavonoids (3). The same appears with HIV, for example, which contained only six papers for the entire period, and one paper was an in silico prediction.

The lack of in vivo studies represents an important limitation to understand the impact of flavonoids on viral infections and the invasion of the CNS. For example, for both the ZIKV and CHIKV, no in vivo studies were found covering this period. These diseases are endemic to tropical third-world countries where accessible therapies are demanding.

Currently, there are models of CNS viral infections that could be handy for studying the pathology of neurotropic viruses and the antiviral effects of flavonoid, with various complexities that comprise, for example, the induction of classical (1) systemic infections [54] or (2) intracerebral infections in both general and stereotactic-specific coordinates [115]. Moreover, it is possible to design (3) transgenic mouse models of the induction of the expression of the viral proteins by the nervous system cells [116] or (4) to administrate the viral protein itself in the brain [117], tools that can help us to understand the molecular mechanisms of damage to the nervous system and the behavioral changes induced by the viral particles and modulated by the flavonoids.

## 7. Conclusions

Considering our 11-year analysis, flavonoids are active against multiple viruses that infect and affect the CNS direct or indirectly, as summarized in the Table 1. Flavonoids present different mechanisms of action against these viruses: viral replication inhibition, direct viral binding, inhibition of viral essential proteins, modulation of the host immune response, and increasing the survival rate of cells and rodents. Although we still consider that there is a lack, especially, of in vivo studies that will allow us to understand the pharmacokinetic properties of flavonoids combined with their mechanism of action in the CNS, the flavonoids are mostly safe compounds with neuroprotective activities and can be considered for main or supplementary adjuvant therapies against neurotrophic viruses.

## Figures and Tables

**Table 1:** Summary of the antiviral activities of the flavonoids most cited in the studies.

Flavonoid	Antiviral Activity	Source	Study Model	Reference
**Baicalin** 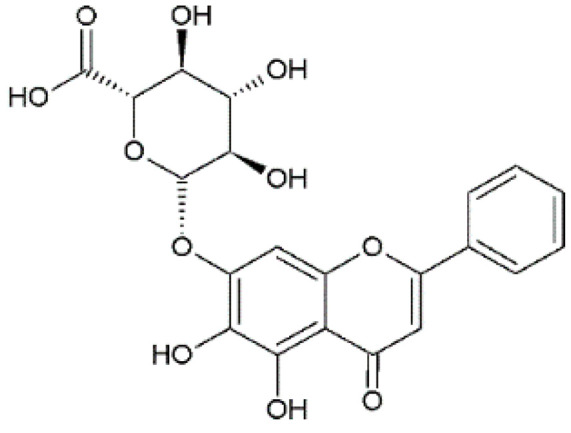	Anti-influenza A H1N1	Synthetic	In vitro/in vivo/in silico	Nayak et al., 2014 [15]
		Isolated from *Radix scutellariae*	In vitro/in vivo	Chu et al., 2015 [16]
		Synthetic	In vitro/in vivo	Li & Wang, 2019 [17]
	Anti-dengue virus (DENV)	Synthetic	In vitro	Moghaddam et al., 2014 [18]
	Anti-enteroviruses	Synthetic	In vitro	Li et al., 2015 [19]
	Anti-Chikungunya virus (CHIKV)	Synthetic	In vitro	Oo et al., 2018 [20]
	Anti-coronaviruses	Synthetic and Bioinformatics	In vitro/In silico	Jo et al., 2020 [21]
**Quercetin** 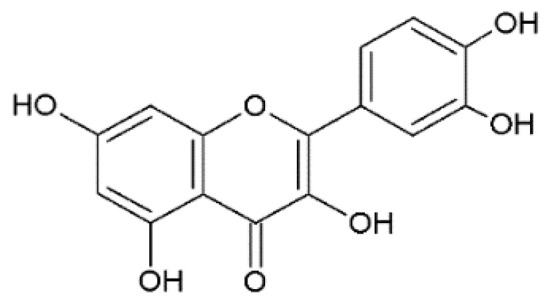	Anti-influenza A H1N1	Synthetic	In vitro	Dayem et al., 2015 [22]
		Bioinformatics	In silico	Sadati et al., 2019 [23]
	Anti-herpes viruses	Synthetic	In vitro	Hung et al., 2015 [24]
	Anti-dengue virus (DENV)	Bioinformatics	In silico	Sarwar et al., 2018 [25]
	Anti-Zika virus (ZIKV)	Synthetic	In vitro	Zou et al., 2020 [26]
**ECGC** 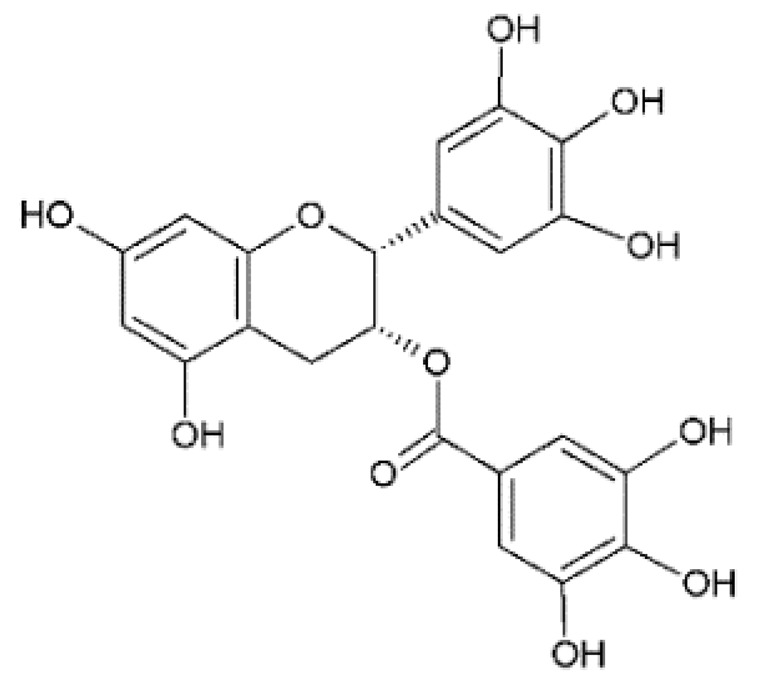	Anti-Chikungunya virus (CHIKV)	Synthetic	In vitro	Weber et al., 2015 [27]
	Anti-Zika virus (ZIKV)	Synthetic	In vitro	Carneiro et al., 2016 [28]
		Synthetic	In vitro	Lim et al., 2017 [29]
		Synthetic	In vitro	Zou et al., 2020 [26]
	Anti-coronaviruses	Synthetic	In vitro	Henss et al., 2021 [30]
**Kaempferol** 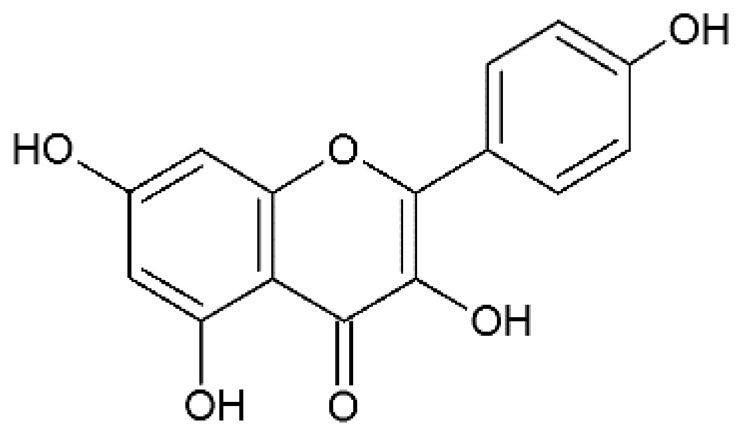	Anti-influenza A H1N1	Synthetic	In vitro	Dayem et al., 2015 [22]
		Bioinformatics	In silico	Sadati et al., 2019 [23]
	Anti-Chikungunya virus (CHIKV) and Anti-dengue virus (DENV)	Bioinformatics	In silico	Keramagi & Skariyachan, 2018 [31]
	Anti-dengue virus (DENV)	Bioinformatics	In silico	Anusuya & Gromiha, 2019 [32]
	Anti-dengue virus (DENV) and Anti-Japanese encephalitis virus (JEV)	Synthetic	In vitro	Care et al., 2020 [33]
**Myricetin** 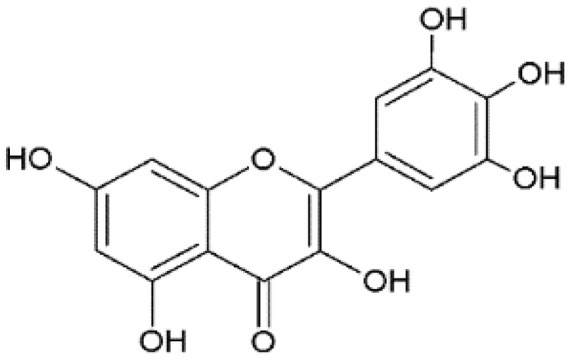	Anti-dengue virus (DENV)	Bioinformatics	In silico	Sarwar et al., 2018 [25]
	Anti-herpes viruses (HSV)	Synthetic	In vitro/in vivo/in silico	Li et al., 2020 [34]
	Anti-Zika virus (ZIKV)	Synthetic	In vitro	Lim et al., 2017 [29]
		Synthetic	In vitro	Zou et al., 2020 [26]
**Rutin** 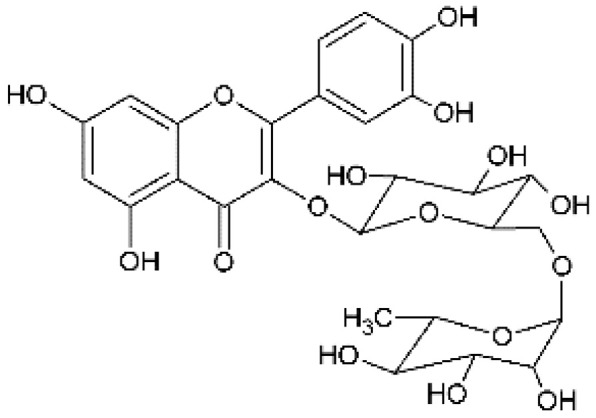	Anti-herpes viruses (HSV)	Synthetic	In vitro	Hung et al., 2015 [24]
	Anti-Zika virus (ZIKV)	Synthetic	In vitro	Lim et al., 2017 [29]
	Anti-influenza A H1N1	Synthetic	In vitro/in vivo	Ling et al., 2020 [35]
**Apigenin** 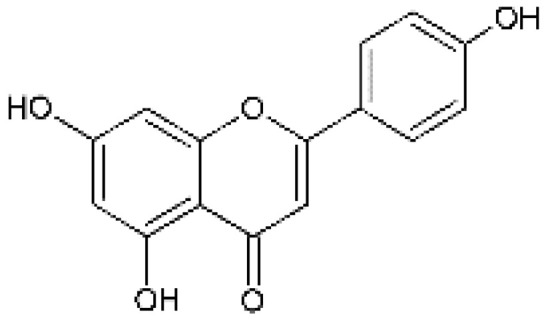	Anti-enteroviruses	Isolated from *Paulownia tomentosa* Steud.	In vitro	Ji et al., 2015 [36]
		Synthetic	In vitro/in vivo	Dai et al., 2019 [37]
	Anti-Chikungunya virus (CHIKV)	Synthetic	In vitro	Pohjala et al., 2011 [38]
**Chrysin** 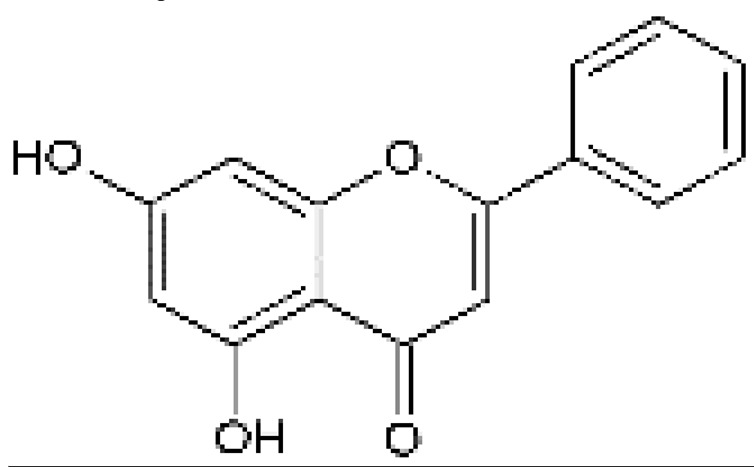	Anti-influenza A H1N1	Bioinformatics	In silico	Sadati et al., 2019 [23]
		Synthetic	In vitro	Kim et al., 2021 [39]
	Anti-Chikungunya virus (CHIKV)	Synthetic	In vitro	Pohjala et al., 2011 [38]

The design of chemical structures of flavonoids was made using the Software ChemSketch V.2021.

## Data Availability

Data sharing not applicable.

## Supplementary Materials

The following supporting information can be downloaded at: https://www.mdpi.com/article/10.3390/ph15091149/s1, Table S1: Summary of all publications included in the study.

## References

[1] Stamatovic S.M., Johnson A.M., Keep R.F., Andjelkovic A.V. (2016). Junctional proteins of the blood-brain barrier: New insights into function and dysfunction. Tissue Barriers.

[2] Jha M.K., Jo M., Kim J.H., Suk K. (2019). Microglia-Astrocyte Crosstalk: An Intimate Molecular Conversation. Neuroscientist.

[3] Agnihotri S.P. (2019). Central Nervous System Opportunistic Infections. Semin. Neurol..

[4] Pal M., Berhanu G., Desalegn C., Kandi V. (2020). Severe Acute Respiratory Syndrome Coronavirus-2 (SARS-CoV-2): An Update. Cureus.

[5] Radzišauskienė D., Vitkauskaitė M., Žvinytė K., Mameniškienė R. (2020). Neurological complications of pandemic A(H1N1)2009pdm, postpandemic A(H1N1)v, and seasonal influenza A. Brain Behav..

[6] Soung A., Klein R.S. (2018). Viral Encephalitis and Neurologic Diseases: Focus on Astrocytes. Trends Mol. Med..

[7] Kaewpoowat Q., Salazar L., Aguilera E., Wootton S.H., Hasbun R. (2016). Herpes simplex and varicella zoster CNS infections: Clinical presentations, treatments and outcomes. Infection.

[8] Van Riel D., Verdijk R., Kuiken T. (2015). The olfactory nerve: A shortcut for influenza and other viral diseases into the central nervous system. J. Pathol..

[9] Wang T., Li Q., Bi K. (2018). shun Bioactive flavonoids in medicinal plants: Structure, activity and biological fate. Asian J. Pharm. Sci..

[10] Costa S.L., Silva V.D.A., Dos Santos Souza C., Santos C.C., Paris I., Muñoz P., Segura-Aguilar J. (2016). Impact of Plant-Derived Flavonoids on Neurodegenerative Diseases. Neurotox. Res..

[11] Kumar S., Pandey A.K., Lu K.P., Sastre J. (2013). Chemistry and Biological Activities of Flavonoids: An Overview. Sci. World J..

[12] Youdim K.A., Qaiser M.Z., Begley D.J., Rice-Evans C.A., Abbott N.J. (2004). Flavonoid permeability across an in situ model of the blood-brain barrier. Free Radic. Biol. Med..

[13] De Boer A.G., Gaillard P.J. (2007). Drug Targeting to the Brain. Annu. Rev. Pharmacol. Toxicol..

[14] Figueira I., Garcia G., Pimpão R.C., Terrasso A.P., Costa I., Almeida A.F., Tavares L., Pais T.F., Pinto P., Ventura M.R. (2017). Polyphenols journey through blood-brain barrier towards neuronal protection. Sci. Rep..

[15] Nayak M.K., Agrawal A.S., Bose S., Naskar S., Bhowmick R., Chakrabarti S., Sarkar S., Chawla-Sarkar M. (2014). Antiviral activity of baicalin against influenza virus H1N1-pdm09 is due to modulation of NS1-mediated cellular innate immune responses. J. Antimicrob. Chemother..

[16] Chu M., Xu L., Zhang M.-B., Chu Z.-Y., Wang Y.-D. (2015). Role of Baicalin in Anti-Influenza Virus A as a Potent Inducer of IFN-Gamma. BioMed Res. Int..

[17] Li R., Wang L. (2019). Baicalin inhibits influenza virus A replication via activation of type I IFN signaling by reducing miR-146a. Mol. Med. Rep..

[18] Moghaddam E., Teoh B.-T., Sam S.-S., Lani R., Hassandarvish P., Chik Z., Yueh A., Abubakar S., Zandi K. (2015). Baicalin, a metabolite of baicalein with antiviral activity against dengue virus. Sci. Rep..

[19] Li X., Liu Y., Wu T., Jin Y., Cheng J., Wan C., Qian W., Xing F., Shi W. (2015). The antiviral effect of baicalin on enterovirus 71 in vitro. Viruses.

[20] Oo A., Rausalu K., Merits A., Higgs S., Vanlandingham D., Bakar S.A., Zandi K. (2018). Deciphering the potential of baicalin as an antiviral agent for Chikungunya virus infection. Antiviral Res..

[21] Jo S., Kim S., Shin D.H., Kim M.-S. (2020). Inhibition of SARS-CoV 3CL protease by flavonoids. J. Enzyme Inhib. Med. Chem..

[22] Dayem A.A., Choi H.Y., Kim Y.B., Cho S.G. (2015). Antiviral effect of methylated flavonol isorhamnetin against influenza. PLoS ONE.

[23] Sadati S.M., Gheibi N., Ranjbar S., Hashemzadeh M.S. (2019). Docking study of flavonoid derivatives as potent inhibitors of influenza H1N1 virus neuraminidas. Biomed. Rep..

[24] Hung P.Y., Ho B.C., Lee S.Y., Chang S.Y., Kao C.L., Lee S.S., Lee C.N. (2015). *Houttuynia cordata* targets the beginning stage of herpes simplex virus infection. PLoS ONE.

[25] Sarwar M.W., Riaz A., Dilshad S.M.R., Al-Qahtani A., Nawaz-Ul-Rehman M.S., Mubin M. (2018). Structure activity relationship (SAR) and quantitative structure activity relationship (QSAR) studies showed plant flavonoids as potential inhibitors of dengue NS2B-NS3 protease. BMC Struct. Biol..

[26] Zou M., Liu H., Li J., Yao X., Chen Y., Ke C., Liu S. (2020). Structure-activity relationship of flavonoid bifunctional inhibitors against Zika virus infection. Biochem. Pharmacol..

[27] Weber C., Sliva K., Von Rhein C., Kümmerer B.M., Schnierle B.S. (2015). The green tea catechin, epigallocatechin gallate inhibits chikungunya virus infection. Antiviral Res..

[28] Carneiro B.M., Batista M.N., Braga A.C.S., Nogueira M.L., Rahal P. (2016). The green tea molecule EGCG inhibits Zika virus entry. Virology.

[29] Lim H., Nguyen T.T.H., Kim N.M., Park J.S., Jang T.S., Kim D. (2017). Inhibitory effect of flavonoids against NS2B-NS3 protease of ZIKA virus and their structure activity relationship. Biotechnol. Lett..

[30] Henss L., Auste A., Schürmann C., Schmidt C., von Rhein C., Mühlebach M.D., Schnierle B.S. (2021). The green tea catechin epigallocatechin gallate inhibits SARS-CoV-2 infection. J. Gen. Virol..

[31] Keramagi A.R., Skariyachan S. (2018). Prediction of binding potential of natural leads against the prioritized drug targets of chikungunya and dengue viruses by computational screening. 3 Biotech.

[32] Anusuya S., Gromiha M.M. (2019). Structural basis of flavonoids as dengue polymerase inhibitors: Insights from QSAR and docking studies. J. Biomol. Struct. Dyn..

[33] Care C., Sornjai W., Jaratsittisin J., Hitakarun A., Wikan N., Triwitayakorn K., Smith D.R. (2020). Discordant activity of kaempferol towards dengue virus and Japanese encephalitis virus. Molecules.

[34] Li W., Xu C., Hao C., Zhang Y., Wang Z., Wang S., Wang W. (2020). Inhibition of herpes simplex virus by myricetin through targeting viral gD protein and cellular EGFR/PI3K/Akt pathway. Antiviral Res..

[35] Ling L., Lu Y., Zhang Y., Zhu H., Tu P., Li H., Chen D. (2020). Flavonoids from *Houttuynia cordata* attenuate H1N1-induced acute lung injury in mice via inhibition of influenza virus and Toll-like receptor signalling. Phytomedicine.

[36] Ji P., Chen C., Hu Y., Zhan Z., Pan W., Li R., Li E., Ge H.M., Yang G. (2015). Antiviral activity of *Paulownia tomentosa* against enterovirus 71 of hand, foot, and mouth disease. Biol. Pharm. Bull..

[37] Dai W., Bi J., Li F., Wang S., Huang X., Meng X., Sun B., Wang D., Kong W., Jiang C. (2019). Antiviral Efficacy of Flavonoids against Enterovirus 71 Infection in Vitro and in Newborn Mice. Viruses.

[38] Pohjala L., Utt A., Varjak M., Lulla A., Merits A., Ahola T., Tammela P. (2011). Inhibitors of alphavirus entry and replication identified with a stable Chikungunya replicon cell line and virus-based assays. PLoS ONE.

[39] Kim S.R., Jeong M.S., Mun S.H., Cho J., Seo M.D., Kim H., Lee J., Song J.H., Ko H.J. (2021). Antiviral activity of chrysin against influenza virus replication via inhibition of autophagy. Viruses.

[40] Hutchinson E.C. (2018). Influenza Virus. Trends Microbiol..

[41] Krammer F. (2015). Emerging influenza viruses and the prospect of a universal influenza virus vaccine. Biotechnol. J..

[42] Van Riel D., Leijten L.M., Verdijk R.M., GeurtsvanKessel C., Van Der Vries E., Van Rossum A.M.C., Osterhaus A.D.M.E., Kuiken T. (2014). Evidence for influenza virus CNS invasion along the olfactory route in an immunocompromised infant. J. Infect. Dis..

[43] Murhekar M., Mehendale S. (2016). The 2015 influenza A (H1N1) pdm09 outbreak in India. Indian J. Med. Res..

[44] Rewar S., Mirdha D., Rewar P. (2015). Treatment and Prevention of Pandemic H1N1 Influenza. Ann. Glob. Health.

[45] Enkhtaivan G., Maria John K.M., Ayyanar M., Sekar T., Jin K.J., Kim D.H. (2015). Anti-influenza (H1N1) potential of leaf and stem bark extracts of selected medicinal plants of South India. Saudi J. Biol. Sci..

[46] Li B., Ni Y., Zhu L.-J., Wu F.-B., Yan F., Zhang X., Yao X.-S. (2015). Flavonoids from Matteuccia struthiopteris and Their Anti-influenza Virus (H1N1) Activity. J. Nat. Prod..

[47] Traboulsi H., Cloutier A., Boyapelly K., Bonin M.A., Marsault É., Cantin A.M., Richter M.V. (2015). The flavonoid isoliquiritigenin reduces lung inflammation and mouse morbidity during influenza virus infection. Antimicrob. Agents Chemother..

[48] Chen L., Dou J., Su Z., Zhou H., Wang H., Zhou W., Guo Q., Zhou C. (2011). Synergistic activity of baicalein with ribavirin against influenza A (H1N1) virus infections in cell culture and in mice. Antiviral Res..

[49] Nair M.S., Huang Y., Fidock D.A., Polyak S.J., Wagoner J., Towler M.J., Weathers P.J. (2021). *Artemisia annua* L. extracts inhibit the in vitro replication of SARS-CoV-2 and two of its variants. J. Ethnopharmacol..

[50] Kashiwada Y., Ahmed F.A., Kurimoto S.I., Kim S.Y., Shibata H., Fujioka T., Takaishi Y. (2012). New α-glucosides of caffeoyl quinic acid from the leaves of *Moringa oleifera* Lam. J. Nat. Med..

[51] Reiberger R., Radilová K., Kráľ M., Zima V., Majer P., Brynda J., Dračínský M., Konvalinka J., Kožíšek M., Machara A. (2021). Synthesis and In Vitro Evaluation of C-7 and C-8 Luteolin Derivatives as Influenza Endonuclease Inhibitors. Int. J. Mol. Sci..

[52] Song J.-M., Lee K.-H., Seong B.-L. (2005). Antiviral effect of catechins in green tea on influenza virus. Antiviral Res..

[53] Cheong Y., Kim M., Ahn J., Oh H., Lim J., Chae W., Yang S.W., Kim M.S., Yu J.E., Byun S. (2021). Epigallocatechin-3-Gallate as a Novel Vaccine Adjuvant. Front. Immunol..

[54] Li M., Wang Y., Jin J., Dou J., Guo Q., Ke X., Zhou C., Guo M. (2021). Inhibitory Activity of Honeysuckle Extracts against Influenza A Virus In Vitro and In Vivo. Virol. Sin..

[55] Li H., Xue Q., Xu X. (2020). Involvement of the Nervous System in SARS-CoV-2 Infection. Neurotox. Res..

[56] Paybast S., Emami A., Koosha M., Baghalha F. (2020). Novel coronavirus disease (COVID-19) and central nervous system complications: What neurologists need to know. Acta Neurol. Taiwan..

[57] Cohen M.E., Eichel R., Steiner-Birmanns B., Janah A., Ioshpa M., Bar-Shalom R., Paul J.J., Gaber H., Skrahina V., Bornstein N.M. (2020). A case of probable Parkinson’s disease after SARS-CoV-2 infection. Lancet Neurol..

[58] Sulzer D., Antonini A., Leta V., Nordvig A., Smeyne R.J., Goldman J.E., Al-Dalahmah O., Zecca L., Sette A., Bubacco L. (2020). COVID-19 and possible links with Parkinson’s disease and parkinsonism: From bench to bedside. npj Parkinson’s Dis..

[59] Taquet M., Geddes J.R., Husain M., Luciano S., Harrison P.J. (2021). 6-month neurological and psychiatric outcomes in 236 379 survivors of COVID-19: A retrospective cohort study using electronic health records. Lancet Psychiatry.

[60] Deng J., Zhou F., Hou W., Silver Z., Wong C.Y., Chang O., Huang E., Zuo Q.K. (2021). The prevalence of depression, anxiety, and sleep disturbances in COVID-19 patients: A meta-analysis. Ann. N. Y. Acad. Sci..

[61] Graham E.L., Clark J.R., Orban Z.S., Lim P.H., Szymanski A.L., Taylor C., DiBiase R.M., Jia D.T., Balabanov R., Ho S.U. (2021). Persistent neurologic symptoms and cognitive dysfunction in non-hospitalized COVID-19 “long haulers”. Ann. Clin. Transl. Neurol..

[62] Park H.R., Yoon H., Kim M.K., Lee S.D., Chong Y. (2012). Synthesis and antiviral evaluation of 7-O-arylmethylquercetin derivatives against SARS-associated coronavirus (SCV) and hepatitis C virus (HCV). Arch. Pharm. Res..

[63] Chang F.R., Yen C.T., Ei-Shazly M., Lin W.H., Yen M.H., Lin K.H., Wu Y.C. (2012). Anti-human coronavirus (anti-HCoV) triterpenoids from the leaves of *Euphorbia neriifolia*. Nat. Prod. Commun..

[64] Jo S., Kim H., Kim S., Shin D.H., Kim M.S. (2019). Characteristics of flavonoids as potent MERS-CoV 3C-like protease inhibitors. Chem. Biol. Drug Des..

[65] Jo S., Kim S., Kim D.Y., Kim M.S., Shin D.H. (2020). Flavonoids with inhibitory activity against SARS-CoV-2 3CLpro. J. Enzyme Inhib. Med. Chem..

[66] Basu A., Sarkar A., Maulik U. (2020). Molecular docking study of potential phytochemicals and their effects on the complex of SARS-CoV2 spike protein and human ACE2. Sci. Rep..

[67] Song J., Zhang L., Xu Y., Yang D., Yang S., Zhang W., Wang J., Tian S., Yang S., Yuan T. (2021). The comprehensive study on the therapeutic effects of baicalein for the treatment of COVID-19 in vivo and in vitro. Biochem. Pharmacol..

[68] Liu H., Ye F., Sun Q., Liang H., Li C., Li S., Lu R., Huang B., Tan W., Lai L. (2021). *Scutellaria baicalensis* extract and baicalein inhibit replication of SARS-CoV-2 and its 3C-like protease in vitro. J. Enzyme Inhib. Med. Chem..

[69] Cao Y., Yang R., Lee I., Zhang W., Sun J., Wang W., Meng X. (2021). Characterization of the SARS-CoV-2 E Protein: Sequence, Structure, Viroporin, and Inhibitors. Protein Sci..

[70] Breitinger U., Ali N.K.M., Sticht H., Breitinger H.G. (2021). Inhibition of SARS CoV Envelope Protein by Flavonoids and Classical Viroporin Inhibitors. Front. Microbiol..

[71] Kornfeind E.M., Visalli R.J. (2018). Human herpesvirus portal proteins: Structure, function, and antiviral prospects. Rev. Med. Virol..

[72] Kukhanova M.K., Korovina A.N., Kochetkov S.N. (2014). Human herpes simplex virus: Life cycle and development of inhibitors. Biochemistry.

[73] Whitley R.J. (2015). Herpes Simplex Virus Infections of the Central Nervous System. Contin. Lifelong Learn. Neurol..

[74] Nicoll M.P., Proença J.T., Efstathiou S. (2012). The molecular basis of herpes simplex virus latency. FEMS Microbiol. Rev..

[75] Vere Hodge R.A., Field H.J. (2013). Antiviral agents for herpes simplex virus. Advances in Pharmacology.

[76] Chen X., Wang Z., Yang Z., Wang J., Xu Y., Tan R., Li E. (2011). *Houttuynia cordata* blocks HSV infection through inhibition of NF-κB activation. Antiviral Res..

[77] Li T., Liu L., Wu H., Chen S., Zhu Q., Gao H., Yu X., Wang Y., Su W., Yao X. (2017). Anti-herpes simplex virus type 1 activity of Houttuynoid A, a flavonoid from *Houttuynia cordata* Thunb. Antiviral Res..

[78] Fukuchi K., Okudaira N., Adachi K., Odai-Ide R., Watanabe S., Ohno H., Yamamoto M., Kanamoto T., Terakubo S., Nakashima H. (2016). Antiviral and antitumor activity of licorice root extracts. In Vivo.

[79] Hu S., Sheng W.S., Schachtele S.J., Lokensgard J.R. (2011). Reactive oxygen species drive herpes simplex virus (HSV)-1-induced proinflammatory cytokine production by murine microglia. J. Neuroinflamm..

[80] Kahrs C.R., Chuda K., Tapia G., Stene L.C., Mårild K., Rasmussen T., Rønningen K.S., Lundin K.E.A., Kramna L., Cinek O. (2019). Enterovirus as trigger of coeliac disease: Nested case-control study within prospective birth cohort. BMJ.

[81] Royston L., Tapparel C. (2016). Rhinoviruses and respiratory enteroviruses: Not as simple as ABC. Viruses.

[82] Goksugur N., Goksugur S. (2010). Images in clinical medicine. Hand, foot, and mouth disease. N. Engl. J. Med..

[83] Wang S.M., Liu C.C., Tseng H.W., Wang J.R., Huang C.C., Chen Y.J., Yang Y.J., Lin S.J., Yeh T.F. (1999). Clinical spectrum of enterovirus 71 infection in children in southern Taiwan, with an emphasis on neurological complications. Clin. Infect. Dis..

[84] Chang L.Y., Lin H.Y., Gau S.S.F., Lu C.Y., Hsia S.H., Huang Y.C., Huang L.M., Lin T.Y. (2019). Enterovirus A71 neurologic complications and long-term sequelae. J. Biomed. Sci..

[85] Maloney J.A., Mirsky D.M., Messacar K., Dominguez S.R., Schreiner T., Stence N.V. (2015). MRI Findings in Children with Acute Flaccid Paralysis and Cranial Nerve Dysfunction Occurring during the 2014 Enterovirus D68 Outbreak. AJNR Am. J. Neuroradiol..

[86] Majer A., McGreevy A., Booth T.F. (2020). Molecular Pathogenicity of Enteroviruses Causing Neurological Disease. Front. Microbiol..

[87] Wang J., Su H., Zhang T., Du J., Cui S., Yang F., Jin Q. (2014). Inhibition of Enterovirus 71 Replication by 7-Hydroxyflavone and Diisopropyl-Flavon7-yl Phosphate. PLoS ONE.

[88] Galochkina A.V., Anikin V.B., Babkin V.A., Ostrouhova L.A., Zarubaev V.V. (2016). Virus-inhibiting activity of dihydroquercetin, a flavonoid from *Larix sibirica*, against coxsackievirus B4 in a model of viral pancreatitis. Arch. Virol..

[89] Davis L.E., Beckham J.D., Tyler K.L. (2008). North American Encephalitic Arboviruses. Neurol. Clin..

[90] Ganesan K., Diwan A., Shankar S.K., Desai S.B., Sainani G.S., Katrak S.M. (2008). Chikungunya encephalomyeloradiculitis: Report of 2 cases with neuroimaging and 1 case with autopsy findings. Am. J. Neuroradiol..

[91] Mehta R., Gerardin P., de Brito C.A.A., Soares C.N., Ferreira M.L.B., Solomon T. (2018). The neurological complications of chikungunya virus: A systematic review. Rev. Med. Virol..

[92] Nelson J., Waggoner J.J., Sahoo M.K., Grant P.M., Pinsky B.A. (2014). Encephalitis caused by Chikungunya virus in a traveler from the Kingdom of Tonga. J. Clin. Microbiol..

[93] Wielanek A.C., De Monredon J., El Amrani M., Roger J.C., Serveaux J.P. (2007). Guillain-Barré syndrome complicating a Chikungunya virus infection. Neurology.

[94] Mota M.T.d.O., Estofolete C.F., Zini N., Terzian A.C.B., Gongora D.V.N., Maia I.L., Nogueira M.L. (2017). Transverse Myelitis as an Unusual Complication of Dengue Fever. Am. J. Trop. Med. Hyg..

[95] Sejvar J.J. (2018). Zika Virus and Other Emerging Arboviral Central Nervous System Infections. Contin. Lifelong Learn. Neurol..

[96] Bollati M., Alvarez K., Assenberg R., Baronti C., Canard B., Cook S., Coutard B., Decroly E., de Lamballerie X., Gould E.A. (2010). Structure and functionality in flavivirus NS-proteins: Perspectives for drug design. Antiviral Res..

[97] Chen S., Wu Z., Wang M., Cheng A. (2017). Innate immune evasion mediated by flaviviridae non-structural proteins. Viruses.

[98] Bakar F.A., Ng L.F.P. (2018). Nonstructural proteins of alphavirus—Potential targets for drug development. Viruses.

[99] Narwal M., Singh H., Pratap S., Malik A., Kuhn R.J., Kumar P., Tomar S. (2018). Crystal structure of chikungunya virus nsP2 cysteine protease reveals a putative flexible loop blocking its active site. Int. J. Biol. Macromol..

[100] Bastos M.d.S., Martins V.d.C.A., da Silva N.L., Jezine S., Pinto S., Aprigio V., Monte R.L., Fragoso S., Puccioni-Sohler M. (2019). Importance of cerebrospinal fluid investigation during dengue infection in Brazilian Amazonia Region. Mem. Inst. Oswaldo Cruz.

[101] Kiat T.S., Pippen R., Yusof R., Ibrahim H., Khalid N., Rahman N.A. (2006). Inhibitory activity of cyclohexenyl chalcone derivatives and flavonoids of fingerroot, *Boesenbergia rotunda* (L.), towards dengue-2 virus NS3 protease. Bioorg. Med. Chem. Lett..

[102] Frabasile S., Koishi A.C., Kuczera D., Silveira G.F., Verri W.A., Dos Santos C.N.D., Bordignon J. (2017). The citrus flavanone naringenin impairs dengue virus replication in human cells. Sci. Rep..

[103] Zandi K., Lim T.-H., Rahim N.-A., Shu M.-H., Teoh B.-T., Sam S.-S., Danlami M.-B., Tan K.-K., Abubakar S. (2013). Extract of *Scutellaria baicalensis* inhibits dengue virus replication. BMC Complement. Altern. Med..

[104] Senthilvel P., Lavanya P., Kumar K.M., Swetha R., Anitha P., Bag S., Sarveswari S., Vijayakumar V., Ramaiah S., Anbarasu A. (2013). Flavonoid from *Carica papaya* inhibits NS2B-NS3 protease and prevents Dengue 2 viral assembly. Bioinformation.

[105] Boonyasuppayakorn S., Saelee T., Visitchanakun P., Leelahavanichkul A., Hengphasatporn K., Shigeta Y., Thanh Huynh T.N., Hann Chu J.J., Rungrotmongkol T., Chavasiri W. (2020). Dibromopinocembrin and Dibromopinostrobin Are Potential Anti-Dengue Leads with Mild Animal Toxicity. Molecules.

[106] Naccache S.N., Thézé J., Sardi S.I., Somasekar S., Greninger A.L., Bandeira A.C., Campos G.S., Tauro L.B., Faria N.R., Pybus O.G. (2016). Distinct zika virus lineage in Salvador, Bahia, Brazil. Emerg. Infect. Dis..

[107] Chiu C.F., Chu L.W., Liao I.C., Simanjuntak Y., Lin Y.L., Juan C.C., Ping Y.H. (2020). The Mechanism of the Zika Virus Crossing the Placental Barrier and the Blood-Brain Barrier. Front. Microbiol..

[108] Gladwyn-Ng I., Cordón-Barris L., Alfano C., Creppe C., Couderc T., Morelli G., Thelen N., America M., Bessières B., Encha-Razavi F. (2017). Stress-induced unfolded protein response contributes to Zika virus–associated microcephaly. Nat. Neurosci..

[109] Jun S.R., Wassenaar T.M., Wanchai V., Patumcharoenpol P., Nookaew I., Ussery D.W. (2017). Suggested mechanisms for Zika virus causing microcephaly: What do the genomes tell us?. BMC Bioinform..

[110] Cataneo A.H.D., Kuczera D., Koishi A.C., Zanluca C., Silveira G.F., de Arruda T.B., Suzukawa A.A., Bortot L.O., Dias-Baruffi M., Verri W.A. (2019). The citrus flavonoid naringenin impairs the in vitro infection of human cells by Zika virus. Sci. Rep..

[111] Mahar Doan K.M., Humphreys J.E., Webster L.O., Wring S.A., Shampine L.J., Serabjit-Singh C.J., Adkison K.K., Polli J.W. (2002). Passive permeability and P-glycoprotein-mediated efflux differentiate central nervous system (CNS) and non-CNS marketed drugs. J. Pharmacol. Exp. Ther..

[112] Jäger A., Saaby L. (2011). Flavonoids and the CNS. Molecules.

[113] Cardona F., Andrés-Lacueva C., Tulipani S., Tinahones F.J., Queipo-Ortuño M.I. (2013). Benefits of polyphenols on gut microbiota and implications in human health. J. Nutr. Biochem..

[114] Leclerc M., Dudonné S., Calon F. (2021). Can Natural Products Exert Neuroprotection without Crossing the Blood–Brain Barrier?. Int. J. Mol. Sci..

[115] Solbrig M.V. (2010). Animal Models of CNS Viral Disease: Examples from Borna Disease Virus Models. Interdiscip. Perspect. Infect. Dis..

[116] Kamitani W., Ono E., Yoshino S., Kobayashi T., Taharaguchi S., Lee B.J., Yamashita M., Kobayashi T., Okamoto M., Taniyama H. (2003). Glial expression of Borna disease virus phosphoprotein induces behavioral and neurological abnormalities in transgenic mice. Proc. Natl. Acad. Sci. USA.

[117] Oh J., Cho W.H., Barcelon E., Kim K.H., Hong J., Lee S.J. (2022). SARS-CoV-2 spike protein induces cognitive deficit and anxiety-like behavior in mouse via non-cell autonomous hippocampal neuronal death. Sci. Rep..

