# Supplementary material for: Pharmacological Potential of Flavonoids against Neurotropic Viruses
Source: Pharmaceuticals (Basel). 2022 Sep 15;15(9):1149. doi: 10.3390/ph15091149 (PMC9502241; doi:10.3390/ph15091149)
Supplement: Supplementary file 1 [file pharmaceuticals-15-01149-s001.zip › pharmaceuticals-1815592-supplementary.pdf]

Pharmacological Potential of Flavonoids against Neurotropic Viruses

Juliana Helena Castro e Silva [1], Jéssica Teles Souza [1], Clarissa Schitine [1], Aníbal de Freitas Santos Júnior [2], Eduardo Muniz Santana Bastos [1] and Silvia Lima Costa [1,*]

[1] Department of Biochemistry and Biophysics, Health Sciences Institute, Federal University of Bahia, Salvador 40110-100, Brazil
[2] Department of Life Sciences, State University of Bahia, Salvador 41150-000, Brazil
* Correspondence: costasl@ufba.br

Table S1. Summary of all publications included in the study.

Author	Article	Country	Year	Journal	Study Model	Flavonoid	Virus
KASHIWADA et al	New a-glucosides of caffeoyl quinic acid from the leaves of Moringa oleifera Lam.	Japan	2011	Journal of natural medicines	*In vitro*	Extract from leaves of Moringa oleifera Lam. (Moringaceae) Ayurvedic medicinal plant.	Influenza A
DOU et al	Effects of Baicalein on Sendai Virus in Vivo Are Linked to Serum Baicalin and Its Inhibition of Hemagglutinin-Neuraminidase	China	2011	Archives of virology	*In vitro/In vivo*	Baicalein	Sendai Virus (SeV)
GOLDWASSER et al	Naringenin Inhibits the Assembly and Long-Term Production of Infectious Hepatitis C Virus Particles Through a PPAR-mediated Mechanism	United States of America	2011	Journal of hepatology	*In vitro*	Naringenin, (WY14, 643, GW9662), and Brefeldin A (BFA)	Hepatitis C virus (HCV)
MEHLA et al	A flavonoid, luteolin, cripples HIV-1 by abrogation of tat function.	United States of America	2011	PLoS One	*In vitro*	Luteolin, Quercetin, and Myricetin	Human Immunodeficiency Virus 1 (HIV-1)
CHEN et al	Synergistic Activity of Baicalein With Ribavirin Against Influenza A (H1N1) Virus Infections in Cell Culture and in Mice	China	2011	Antiviral research	*In vitro/In vivo*	Baicalein	Influenza A (H1N1) virus
SONG et al	Quercetin 7-rhamnoside reduces porcine epidemic diarrhea virus replication via independent pathway of viral induced reactive oxygen species	South Korea	2011	Virology journal	*In vitro*	Quercetin 7-rhamnoside (Q7R)	Porcine epidemic diarrhea virus (PEDV CV)
BACHMETOV et al	Suppression of Hepatitis C Virus by the Flavonoid Quercetin Is Mediated by Inhibition of NS3 Protease Activity	Israel	2011	Journal of viral hepatitis	*In vitro*	Quercetin	Hepatitis C virus (HCV)
OZÇELIK, KARTAL, & ORHAN	Cytotoxicity, antiviral and antimicrobial activities of alkaloids, flavonoids, and phenolic acids	Turkey	2011	Pharmaceutical biology	*In vitro*	Quercetin, Genistein, Apigenin, Naringin, Silibinin, and Silymarin	Virus herpes simplex type 1 and parainfluenza (type-3) virus

YARMOLINSKY et al	Potent Antiviral Flavone Glycosides From Ficus Benjamina Leaves	Israel	2012	Fitoterapia	*In vitro*	Quercetin 3-O-α-rhamnopyranosyl(1—6)-β-glucopyranoside; kaempferol 3-O-α-rhamnopyranosyl(1—6)-β-glucopyranoside; kaempferol 3-O-a-rhamnopyranosyl(1—6)-b-galactopyranoside	Herpes Simplex Virus-1 (HSV-1)
LU & CHONG	Combining Molecular Docking and Molecular Dynamics to Predict the Binding Modes of Flavonoid Derivatives With the Neuraminidase of the 2009 H1N1 Influenza A Virus	China	2012	International journal of molecular sciences	*In silico*	20 flavonoid derivatives (2,3-dihydrobenzofuran and 5,7-dihydroxychromen-4-one backbones) that were constructed and minimized using VEGA ZZ [41] and ISIS/DRAW [42] programs.	Influenza A (H1N1) virus
CHEN et al	Houttuynoids A-E, Anti-Herpes Simplex Virus Active Flavonoids With Novel Skeletons From Houttuynia Cordata	China	2012	Organic letters	*in vitro*	Houttuynoids A E (1 5)	Anti-Herpes Simplex Virus
PARK et al	Synthesis and Antiviral Evaluation of 7-O-arylmethylquercetin Derivatives Against SARS-associated Coronavirus (SCV) and Hepatitis C Virus (HCV)	Korea	2012	Archives of pharmacal research	*In vitro*	26 aromatic substituents of 7-O-arylmethylquercetin derivatives	SARS-associated coronavirus (SARS-CoV, SCV) and hepatitis C virus (HCV)
HAYASHI et al	In vitro and in vivo evaluation of a novel antiherpetic flavonoid, 4'-phenylflavone, and its synergistic actions with acyclovir.	Japan	2012	Archives of virology	*In vitro/In vivo*	4'-Phenylflavone	Herpes simplex virus 1/2 (HSV-1 and HSV-2), poliovirus type 1; human cytomegalovirus (HCMV); and Influenza A virus

HAID et al	A Plant-Derived Flavonoid Inhibits Entry of All HCV Genotypes Into Human Hepatocytes	Germany	2012	Gastroenterolog y	*In vitro*	Synthetic Ladanein (BJ486K) produced in laboratory and extracts from Marrubium peregrinum L (Lamiaceae)	Hepatitis C virus (HCV)
WANG et al	[Protein Kinase Inhibitor Flavopiridol Inhibits the Replication of Influenza Virus in Vitro]	China	2012	Acta microbiologica Sinica	*In vitro*	Flavopiridol	Influenza Virus
MA et al	Anti-hepatitis B Virus Activity of Chickweed [Stellaria Media (L.) Vill.] Extracts in HepG2.2.15 Cells	China	2012	Molecules	*In vitro*	Extract from Stellaria media (L.) Vill., a Chinese folk medicine that belongs to the Caryophyllaceae flowering plant family.	Hepatitis B virus (HBV)
CONTIN et al	Eight Flavonoids and Their Potential as Inhibitors of Human Cytomegalovirus Replication	France	2012	Antiviral research	*In vitro*	Baicalein, naringenin, quercetin and quercetagetin (flavones, flavanones and flavonols, respectively), chalcones (20,30,40-trihydroxychalcone, 2,20,40-trihydroxychalcone, naringenin chalcone and Butein) were produced by EA4021 (Limoges, France)	Cytomegalovirus (HCMV)
LIU et al	Discovery of Flavonoid Derivatives as anti-HCV Agents via Pharmacophore Search Combining Molecular Docking Strategy	China	2012	European journal of medicinal chemistry	*In silico/In vitro*	Apigenin and Luteolin	Hepatitis C virus (HCV)
CALLAND et al	(-)-Epigallocatechin-3-gallate Is a New Inhibitor of Hepatitis C Virus Entry	France	2012	Hepatology (Baltimore, Md.)	*In vitro*	(-)-epigallocatechin-3-gallate (EGCG) , (+)-catechin, (−)-epicatechin (EC), (−)-epicatechin-3-gallate (ECG), (−)-	Hepatitis C virus (HCV)

						epigallocatechin (EGC), and EGCG	
VISINTINI-JAIME et al	In Vitro Antiviral Activity of Plant Extracts From Asteraceae Medicinal Plants	Argentina	2013	Virology journal	*In vitro*	Organic (OE) and aqueous extracts (AE) from: Baccharis gaudichaudiana, Baccharis spicata, Bidens subalternans, Pluchea sagittalis, Tagetes minuta and Tessaria absinthioides, all medicinal plants belonging to the Asteraceae family.	Bovine viral diarrhea virus, herpes simplex virus type 1 (HSV-1), poliovirus type 2 (PV-2) and vesicular tomatitis virus
CHERRY et al	Structure Based Identification and Characterization of Flavonoids That Disrupt Human papillomavirus-16 E6 Function	United States of America	2013	PloS one	*In silico/In vitro*	Flavonoid luteolin and the novel flavone analog CAF-24	Human papillomavirus (HPV)
KRALJ et al	Development of Flavonoid-Based Inverse Agonists of the Key Signaling Receptor US28 of Human Cytomegalovirus	Germany	2013	Journal of medicinal chemistry	*In vitro*	A series of 31 chalcone- and flavonoid-based derivatives	Human cytomegalovirus (HCMV)
KIM et al	Inhibition of Influenza Virus Internalization by (-)-epigallocatechin-3-gallate	South Korea	2013	Antiviral research	*In vitro*	(-)-Epigallocatechin-3-gallate (EGCG),epigallocatechin (EGC), epicatechin monogallate (ECG), and 2' 2'-bisepigallocatechin digallate (bEGCdG),	Influenza virus
CHEN et al	Anti HSV-1 Flavonoid Derivatives Tethered With Houttuynin From Houttuynia Cordata	China	2013	Planta medica	*In vitro*	Extract from Houttuynia cordata Thunb. (Saururaceae) widely cultivated or harvested in China and other Asian countries (collected in Guangdong province in July 2009).	Herpes simplex virus 1 (HSV-1)

COULERIE et al	Structure-activity Relationship Study of Biflavonoids on the Dengue Virus Polymerase DENV-NS5 RdRp	France	2013	Planta medica	*In vitro*	Amentoflavone, Podocarpusflavone, Isoginkgetin, Hinokiflavone derivative from Dacrydium balansae (Podocarpaceae) and robustaflavone, Sequoiaflavone, Bilobetin, Sotetsuflavone, Ginkgetin, Sciadopitysin isolated from D. araucarioides (Podocarpaceae from South province of New Caledonia), and apigenin derivatives (Acacetin, genkwanin, 7,4′-dimethylapigenin , trimethylapigenin ,pinocembrin, naringenin, galangin, quercetin, rhamnetin, and kaempferol).	Dengue virus (DENV)
SENTHILVEL et al	Flavonoid From Carica Papaya Inhibits NS2B-NS3 Protease and Prevents Dengue 2 Viral Assembly	India	2013	Bioinformation	*In silico*	Anti-dengue activities of the extracts from Carica papaya (family Caricaceae). Seven phenolic compounds: quercetin, protocatechuic acid, p-coumaric acid, caffeic acid, chlorogenic acid, kaempferol, and 5,7-dimethoxycoumarin.	Dengue 2 virus (DENV-2).

ZANDI et al	Extract of Scutellaria Baicalensis Inhibits Dengue Virus Replication	Malaysia	2013	BMC complementary and alternative medicine	*In vitro*	Aqueous extract of the roots of Scutellaria baicalensis (S. baicalensis)	Dengue virus (DENV-1, DENV-2, DENV-3 and DENV-4) serotypes.
LOU et al	Inhibition of Hepatitis C Virus Replication in Vitro by Xanthohumol, a Natural Product Present in Hops	China	2013	Planta medica	*In vitro*	Xanthohumol (XN)	Hepatitis C virus
LI et al	Design and Discovery of Flavonoid-Based HIV-1 Integrase Inhibitors Targeting Both the Active Site and the Interaction With LEDGF/p75	China	2014	Bioorganic & medicinal chemistry	*In silico/In vitro*	Syntheses to generate a series 68 mono-substituted flavonoid derivatives (including quercetin, baicalein, luteolin, apigenin, crysin, naringenin, genistein)	Human Immunodeficiency Virus 1 (HIV-1)
ZHANG et al	The Flavonoid From Polygonum Perfoliatum L. Inhibits Herpes Simplex Virus 1 Infection	China	2014	Acta virologica	*In vitro/In vivo*	Extract from Polygonum perfoliatum L (commonly known as mile-a-minute weed).	Herpes simplex virus 1 (HSV-1)
YIN et al	Antiviral Activity of Total Flavonoid Extracts From Selaginella Moellendorffii Hieron Against Coxsackie Virus B3 In Vitro and In Vivo	China	2014	Evidence-based complementary and alternative medicine : eCAM	*In vitro/In vivo*	Extract from The whole herbs of Selaginella moellendorffii Hieron.	Coxsackie virus B3 (CVB3)
JOHN et al	Metabolic Variations, Antioxidant Potential, and Antiviral Activity of Different Extracts of Eugenia Singampattiana (An Endangered Medicinal Plant Used by Kani Tribals, Tamil Nadu, India) Leaf	South Korea	2014	BioMed research international	*In vitro*	Extract from Eugenia singampattiana is a small tree belonging to the family Myrtaceae.	Porcine reproductive and respiratory syndrome virus (PRRSV)
DOS SANTOS et al	Quercetin and Quercetin 3-O-glycosides From Bauhinia Longifolia (Bong.) Steud. Show anti-Mayaro Virus Activity	Brazil	2014	Parasites & vectors	*In vitro*	Extract from of Bauhinia longifolia (Bong.) Steud.	Mayaro virus (MAYV)

NAYAK et al	Antiviral Activity of Baicalin Against Influenza Virus H1N1-pdm09 Is Due to Modulation of NS1-mediated Cellular Innate Immune Responses	India	2014	The Journal of antimicrobial chemotherapy	*In vitro/in silico/in vivo*	Baicalin	Influenza A (H1N1) virus
HOSSAIN et al	Antiviral Activity of 3,4'-dihydroxyflavone on Influenza a Virus	South Korea	2014	Journal of microbiology (Seoul, Korea)	*In vitro/In vivo*	3-hydroxyflavone (3-HF), 3,2'-dihydroxyflavone (3,2'-DHF), 3,3'-dihydroxyflavone (3,3'-DHF), 3,4'-dihydroxyflavone (3,4'-DHF), 4-hydroxyflavone (4-HF), 4'-hydroxy-5-methoxyflavone (4'-H5-MF), 4'-hydroxy-6-methoxyflavone (4'-H6-MF), 4'-hydroxy-7-methoxyflavone (4'-H7-MF), 5,7-dihydroxy-3',4',5'-trimethoxyflavone (5,7-D,3',4',5'-TMF), 6,4'-dihydroxyflavone (6,4'-DHF), 7,8,4'-trihydroxyflavone (7,8,4'-THF), 3,5,7-trihydroxy-4'-methoxyflavone (diosmetin), and 3,5,7-3',4'-pentahydroxyflavone (quercetin),	Influenza A (H1N1) virus
MICHAELIS et al	Effects of Flavonoid-Induced Oxidative Stress on anti-H5N1 Influenza a Virus Activity Exerted by Baicalein and Biochanin A	Germany	2014	BMC research notes	*In vitro*	Biochanin A and Baicalein	Influenza A (H5N1) virus
MOGHADDAM et al	Baicalin, a Metabolite of Baicalein With Antiviral Activity Against Dengue Virus	Malaysia	2014	Scientific reports	*In vitro*	Baicalin	Dengue virus (DENV)

WANG et al	Anti-enterovirus 71 Effects of Chrysin and Its Phosphate Ester	China	2014	PloS one	*In silico/In vitro*	Chrysin (5, 7-dihydroxyflavone, C15H10O4, Mr: 254.24), kaempferol (Kae, C15H10O6, Mr: 286.23) and diisopropyl chrysin-7-yl phosphate (CPI)	Enterovirus 71 (EV71)
ZHANG et al	Apigenin Inhibits enterovirus-71 Infection by Disrupting Viral RNA Association With Trans-Acting Factors	China	2014	PloS one	*In vitro*	Apigenin, Kaempferol, Hesperetin, Naringenin	Enterovirus 71 (EV71)
HUANG et al	(-)-Epigallocatechin-3-gallate Inhibits Entry of Hepatitis B Virus Into Hepatocytes	Taiwan	2014	Antiviral research	*In vitro*	(-)-Epigallocatechin-3-gallate	Hepatitis B virus (HBV)
SHIBATA et al	The Flavonoid Apigenin Inhibits Hepatitis C Virus Replication by Decreasing Mature microRNA122 Levels	Japan	2014	Virology	*In vitro*	(+)-Catechin (C), (-)-epicatechin (EC), (-)-epigallocatechin (EGC), (-)-epicatechin-3-gallate (ECG), (-)-epigallocatechin-3-gallate (EGCG), rutin, quercetin, chrysi	Hepatitis C virus (HCV)
WANG et al	Inhibitory Effects of Pinus Massoniana Bark Extract on Hepatitis C Virus in Vitro	China	2015	Pharmaceutical biology	*In vitro*	Extract from Pinus massoniana Lamb (Pinaceae).	Hepatitis C virus (HCV)
SONG et al	Antiviral Activity of Chrysin Derivatives Against Coxsackievirus B3 in Vitro and in Vivo	South Korea	2015	Biomolecules & therapeutics	*In vitro/In vivo*	Chysin and synthesis of chrysin derivatives (2-11)	Coxsackievirus B3 (CVB3) enterovirus
JI et al	Antiviral Activity of Paulownia Tomentosa Against Enterovirus 71 of Hand, Foot, and Mouth Disease	China	2015	Biological & pharmaceutical bulletin	*In vitro*	Apigenin, naringenin and quercetin and extract from Paulownia tomentosa (THUNB.) STEUD.	Enterovirus 71 (EV71) and coxsackievirus A16 (CAV16)
TRABOULSI et al	The Flavonoid Isoliquiritigenin Reduces Lung Inflammation and Mouse Morbidity During Influenza Virus Infection	Canada	2015	Antimicrobial agents and chemotherapy	*In vitro/In vivo*	Isoliquiritigenin ILG and ILG-p (fosfato) were synthesized by our group.	Influenza A (H1N1) virus

ABDAL et al	Antiviral Effect of Methylated Flavonol Isorhamnetin Against Influenza	South Korea	2015	PloS one	*In vitro/In vivo*	Quercetin, kaempferol, isorhamnetin, diosmetin, and eriodictyol	Influenza virus A (H1N1)
LI et al	The Antiviral Effect of Baicalin on Enterovirus 71 In Vitro	China	2015	Viruses	*In vitro*	Baicalin	Enterovirus 71 (EV71)
VARGAS et al	A Network Flow Approach to Predict Protein Targets and Flavonoid Backbones to Treat Respiratory Syncytial Virus Infection	Brazil	2015	BioMed research international	*In silico*	Resveratrol, quercetin, tricetin, apigenin, and myricetin	Respiratory syncytial virus (RSV
CALLAND et al	Polyphenols Inhibit Hepatitis C Virus Entry by a New Mechanism of Action	France	2015	Journal of virology	*In vitro*	Epigallocatechin-3-gallate (EGCG), Gallocatechin-3-gallate (GCG), Delphinidin chloride, cyanidin chloride, myrtillin chloride, pelargonidin chloride, tricetinidin chloride, myricetin, and petunidin chloride.	Hepatitis C virus (HCV)
MATHEW et al	Computational Docking Study of p7 Ion Channel From HCV Genotype 3 and Genotype 4 and Its Interaction With Natural Compounds	Saudi Arabia	2015	PloS one	*In silico*	Epigallocatechin-3-gallate, Apigenin, Naringenin, Luteolin, Quercetin, Ladanein, and Silymarin.	Hepatitis C virus (HCV)
RAJ et al	Flavonoids as Multi-target Inhibitors for Proteins Associated With Ebola Virus: In Silico Discovery Using Virtual Screening and Molecular Docking Studies	India	2015	Interdisciplinary sciences, computational life sciences	*In silico*	Compound id's named ST059622 (Gossypetin), ST50903219, ST50940361, ST101866, ST078351 and ST060285 (Taxifolin)	Ebola virus
ENKHTAIVAN et al	Anti-influenza (H1N1) Potential of Leaf and Stem Bark Extracts of Selected Medicinal Plants of South India	South Korea	2015	Saudi journal of biological sciences	*In vitro*	Extracts from Strychnos minor, Diotacanthus albiflorus, Strychnos nux-vomica, Chloroxylon swietenia, and Cayratia pedata.	Influenza A (H1N1) virus

QIAN et al	Apigenin Restricts FMDV Infection and Inhibits Viral IRES Driven Translational Activity	China	2015	Viruses	*In vitro*	Apigenin, baicalein, chrysin, liquiritigenin, quercetin, kaempferol and galangin.	Foot-and-mouth virus
THIRUVENGADAM et al	Induction of hairy roots by Agrobacterium rhizogenes-mediated transformation of spine gourd (Momordica dioica Roxb. ex. willd) for the assessment of phenolic compounds and biological activities	South Korea	2015	Scientia horticulturae	*In vitro*	Extract from Momordica dioica Roxb. ex. Willd (Family: Cucurbitaceae).	IBD virus
CHU et al	Role of Baicalin in Anti-Influenza Virus A as a Potent Inducer of IFN-Gamma	China	2015	BioMed research international	*In vitro/in vivo*	Baicalin	Influenza A (H1N1) virus
LIPSON et al	Comparison of α-Glucosyl Hesperidin of Citrus Fruits and Epigallocatechin Gallate of Green Tea on the Loss of Rotavirus Infectivity in Cell Culture	United States of America	2015	Frontiers in microbiology	*In vitro*	(–)-EGCG (epigallocatechin gallate) And semisynthetic α-glucosyl hesperitin (GH)	Rotavirus
LI et al	Flavonoids From Matteuccia Struthiopteris and Their Anti-influenza Virus (H1N1) Activity	China	2015	Journal of natural products	*In vitro*	Extract from The rhizomes of Matteuccia struthiopteris (L.) Todar (Onocleaceae).	Influenza A (H1N1) virus
ZHONG et al	Discovery of Metal Ions Chelator Quercetin Derivatives With Potent Anti-HCV Activities	China	2015	Molecules (Basel, Switzerland)	*In silico/In vitro*	38 newly synthesized compounds, includin quercetin (quercetin analogues)	Hepatitis C virus (HCV)
FUKUCHI et al	Antiviral and Antitumor Activity of Licorice Root Extracts	Japan	2016	In vivo (Athens, Greece)	*In vitro*	Flavonoid and chalcone derivatives (liquiritin apioside, liquiritigenin 7-apiosylglucoside, liquiritin, neoliquiritin, liquiritigenin, isoliquiritin apioside, licurazid, isoliquiritin, neoisoliquiritin, isoliquiritigenin); extracts of licorice root	Human immunodeficiency virus (HIV) or herpes simplex virus (HSV)

						and flavonoid-rich fraction of water extract of liquorice root.	
QIU et al	Prophylactic Efficacy of Quercetin 3-β-O-d-Glucoside Against Ebola Virus Infection	Canada	2016	Antimicrobial agents and chemotherapy	*In vitro/in vivo*	Quercetin 3-β-O-d-glucoside (Q3G)	Ebola virus, Sudan virus (SUDV), and Reston virus (RESTV)
BAKR et al	Phenolic profile of Centaurea aegyptiaca l. Growing in egypt and its cytotoxic and antiviral activities	Egypt	2016	African journal of traditional, complementary, and alternative medicines : AJTCAM	*In vitro*	Extract from Aerial parts of Centaurea aegyptiaca L (Centaurea, the fourth biggest genus in the family Asteraceae).	Hepatitis A virus (HAV); Herpes simplex virus type 1 (HSV 1).
DERKSEN et al	Antiviral Activity of Hydroalcoholic Extract From Eupatorium Perfoliatum L. Against the Attachment of Influenza A Virus	Germany	2016	Journal of ethnopharmacology	*In vitro*	Rutin; Hyperoside; Isoqurcitrin; Trifolin; Astragalin; Eupafolin and Extract and fraction-rich from Dried Eupatorium perfoliatum herb (Lot 3458).	Influenza A (H1N1) virus
ÜRMÉNYI et al	Anti-HSV-1 and HSV-2 Flavonoids and a New Kaempferol Triglycoside From the Medicinal Plant Kalanchoe Daigremontiana	Brazil	2016	Chemistry & biodiversity	*In vitro*	Kaempferol-3-O-β-D-xylopyranosyl (1 →2)-α-L-rhamnopyranoside, Kd-AC-5-3 (ethyl acetate fraction); (1) Quercetin-3-O-β-D-xylopyranosyl (1→2)- α-L-rhamnopyranoside,	Herpes simplex virus types 1 and 2 (HSV-1 and HSV-2)

KARIMI et al	Anti-adenovirus Activity, Antioxidant Potential, and Phenolic Content of Black Tea (Camellia Sinensis Kuntze) Extract	Iran	2016	Journal of complementary & integrative medicine	*In vitro*	Black tea from *Camellia sinensis.*	Adenovirus (ADV)
FAN et al	Antiviral Activity of Luteolin Against Japanese Encephalitis Virus	China	2016	Virus research	*In vitro*	Luteolin	Japanese encephalitis virus (JEV)
GRIENKE et al	Discovery of Prenylated Flavonoids With Dual Activity Against Influenza Virus and Streptococcus Pneumoniae	Austria	2016	Scientific reports	*In vitro*	Sanggenon B, sanggenon C, sanggenon D, sanggenon G, sanggenol A, kuwanon L (6), and the 1:1 mixture of moracin O and moracin P	Influenza A (H1N1) virus
DU et al	Inhibition of Dengue Virus Replication by Diisopropyl chrysin-7-yl Phosphate	China	2016	Science China. Life sciences	*In vitro*	Diisopropylchrysin-7-yl phosphate (CPI)	Dengue virus (DENV)
GALOCHKINA et al	Virus-inhibiting Activity of Dihydroquercetin, a Flavonoid From Larix Sibirica, Against Coxsackievirus B4 in a Model of Viral Pancreatitis	Russia	2016	Archives of virology	*In vitro/in vivo*	Dihydroquercetin (taxifolin, DHQ) (2,3-dihydro-3,5,7-trihydroxy-2-(3,4-dihydroxyphenyl)-4H-1-benzopyran-4-one	Coxsackie virus B4 (CVB4)
ROJAS et al	Effect of Quercetin on Hepatitis C Virus Life Cycle: From Viral to Host Targets	Spain	2016	Scientific reports	*In vitro*	Quercetin	Hepatitis C virus (HCV)
WU et al	Luteolin Inhibits Epstein-Barr Virus Lytic Reactivation by Repressing the Promoter Activities of Immediate-Early Genes	Taiwan	2016	Antiviral research	*In vitro*	Luteolin	Epstein-Barr virus (EBV)
AURORI et al	Bay Laurel (Laurus Nobilis) as Potential Antiviral Treatment in Naturally BQCV Infected Honeybees	Romania	2016	Virus research	*In vitro/in vivo*	L. nobilis ethanolic extracts. Dry Laurus nobilis leaves of Greek origin.	Black queen cell virus (BQCV)
KERL et al	Total Synthesis of the Antiviral Natural Product Houttuynoid B	Germany	2016	Chemistry (Weinheim an der Bergstrasse, Germany)	*In silico*	Synthesis of houttuynoid B	Herpes simplex virus

TSUKADA et al	A new class of hepatitis B and D virus entry inhibitors, proanthocyanidin and its analogs, that directly act on the viral large surface proteins.	Japan	2017	Hepatology	*In vitro*	Procyanidin B1, Proanthocyanidin, Oolonghomobisflavan C	Hepatitis B virus (HBV)
CHEN et al	Treatment effect of a flavonoid prescription on duck virus hepatitis by its hepatoprotective and antioxidative ability.	China	2017	Pharm. Biol.	*In vivo/in vitro*	Baicalin, linarin, icariin and notoginsenoside R1	Duck Hepatitis A Virus Type 1 (DHAV-1)
FRABASILE et al	The citrus flavanone naringenin impairs dengue virus replication in human cells.	Brazil	2017	Scientific Reports	*In vitro*	Naringenin	Dengue virus (DENV)
CHEN et al	Anti-DHAV-1 reproduction and immuno-regulatory effects of a flavonoid prescription on duck virus hepatitis.	China	2017	Pharm. Biol.	*In vitro/in vivo*	Baicalin, linarin, icariin and notoginsenoside R1	Duck Hepatitis A Virus Type 1 (DHAV-1)
SEO & CHOI et al	Inhibitory mechanism of five natural flavonoids against murine norovirus.	South Korea	2017	Phytomedicine	*In vitro*	Epigallocatechin gallate (EGCG), epicatechin gallate (ECG), quercetin, daidzein, fisetin	Murine norovirus
LI et al	Anti-herpes simplex virus type 1 activity of Houttuynoid A, a flavonoid from Houttuynia cordata Thunb.	China	2017	Antiviral Research	*In vitro/in vivo*	Houttuynoid and extracts from Houttuynia cordata Thunb. (Saururaceae)	Herpes Simplex Virus-1 (HSV-1)
DOS SANTOS et al	In vitro antiherpes effect of C-glycosyl flavonoid enriched fraction of Cecropia glaziovii encapsulated in PLGA nanoparticles.	Brazil	2017	Mater Sci Eng C Mater Biol Appl.	*In vitro*	Freeze-dried enriched flavonoid fraction	Herpes simplex virus 1 (HSV-1)
BANG et al	Anti-influenza effect of the major flavonoids from Salvia plebeia R.Br. via inhibition of influenza H1N1 virus neuraminidase.	South Korea	2018	Natural Products Research	*In vitro*	6-hydroxyluteolin 7-O-β-d-glucoside (1), nepitrin (2), homoplantaginin (3)	Influenza A (H1N1) virus

BRANDÃO et al	Antiviral Activity of Fridericia formosa (Bureau) L. G. Lohmann (Bignoniaceae) Extracts and Constituents.	Brazil	2017	Journal of Tropical Medicine	*In vitro*	Mangiferin (1; 2-β-D-Glucopyranosyl-1,3,6,7-tetrahydroxy-9H-xanthen-9-one). 2′-O-trans-caffeoylmangiferin (2; 2-(2′-O-trans-caffeoyl)-C-β-D-glucopyranosyl-1,3,6,7-tetrahydroxyxanthone). 2′-O-trans-coumaroylmangiferin (3; 2-(2′-O-trans-coumaroyl)-C-β-D-glucopyranosyl-1,3,6,7-tetrahydroxyxanthone). Chrysin (4; 5,7-Dihydroxy-2-phenyl-4H-1-benzopyran-4-one). 2′-O-trans-cinnamoylmangiferin (5; 2-(2′-O-trans-cinnamoyl)-C-β-D-glucopyranosyl-1,3,6,7-tetrahydroxyxanthone);	Herpes Simplex Virus-1 (HSV-1)
BOSE et al	Identification of a flavonoid isolated from plum (Prunus domestica) as a potent inhibitor of Hepatitis C virus entry.	India	2017	Scientific Reports	*In vitro/ex vivo*	Rutin and different crude extracts from plum (*Prunus domestica*).	Hepatitis C virus (HCV)
HUH et al	C-Methylated Flavonoid Glycosides from Pentarhizidium orientale Rhizomes and Their Inhibitory Effects on the H1N1 Influenza Virus.	South Korea	2017	Journal of Natural Products	*In vitro*	13 C-methylated flavonoid glycosides (1−13), along with 15 previously known flavonoids (14–28), (including naringenin)	Influenza A (H1N1)
ORTEGA et al	The role of the glycosyl moiety of myricetin derivatives in anti-HIV-1 activity in vitro.	Venezuela	2017	AIDS Res Ther.	*In vitro*	Myricetin 3-rhamnoside and myricetin 3-(6-rhamnosylgalactoside)	Human immunodeficiency virus 1 (HIV-1)

JIN et al	Oroxylin A suppresses influenza A virus replication correlating with neuraminidase inhibition and induction of IFNs.	China	2018	Biomed Pharmacother. 2	*In vitro*	Oroxylin A	Influenza A
LI et al	Houttuynoid M, an Anti-HSV Active Houttuynoid from Houttuynia cordata Featuring a Bis-houttuynin Chain Tethered to a Flavonoid Core.	China	2017	Journal of Natural Products	*In vitro/in vivo*	Houttuynoid M (1) and houttuynoid A (2)	Herpes Simplex virus (HSV-2)
SHIMIZU et al	Flavonoids from Pterogyne nitens Inhibit Hepatitis C Virus Entry.	Brazil	2017	Scientific Reports	*In vitro*	Sorbifolin (1) and pedalitin (2)	Hepatitis C virus (HCV)
LIM et al	Inhibitory effect of flavonoids against NS2B-NS3 protease of ZIKA virus and their structure activity relationship	South Korea	2017	Biotechnol Lett	*In vitro*	22 flavonoids	Zika Virus (ZIKV)
SCHIAVONI et al	CD38 modulates respiratory syncytial virus-driven proinflammatory processes in human monocyte-derived dendritic cells.	Italy	2018	Immunology	*In vitro*	Kuromanin	Human respiratory syncytial virus type A2 (RSV)
KANNAN & KOLANDAIVEL	The inhibitory performance of flavonoid cyanidin-3-sambubiocide against H274Y mutation in H1N1 influenza virus.	India	2018	J biomol struct dyn	*In silico*	Cyanidin-3-sambubiocid	Influenza A (H1N1) virus
MIN et al	A flavonoid compound library screen revealed potent antiviral activity of plant-derived flavonoids on human enterovirus A71 replication.	Singapore	2018	Antiviral Research	*In vitro*	ST077124 (10mg) and ST024734 (10mg)	Enterovirus 71 (EV71)
ANUSUYA & GROMIHA	Structural basis of flavonoids as dengue polymerase inhibitors: insights from QSAR and docking studies.	Japan	2019	J biomol struct dyn	*In silico*	33 flavonoids	Dengue virus (DENV)
VALENZUELA et al	Alpinone exhibited immunomodulatory and antiviral activities in Atlantic salmon.	Chile	2018	Fish Shellfish Immunol.	*In vitro*	Alpinone and Pinocembrine	Infectious Salmon Anemia virus.
LIU et al	In vitro anti-influenza virus effect of total flavonoid from Trollius ledebouri Reichb.	China	2018	J Int Med Res	*In vitro*	Extract from Trollius ledebouri Reichb (TFTLR).	Influenza A (H1N1 and H3N2) virus
FERREIRA et al	Detection of the antiviral activity of epicatechin isolated from Salacia crassifolia (Celastraceae) against Mayaro virus based on protein C homology modelling and virtual screening.	Brazil	2018	Archives of Virology	*In silico/In vitro*	Epicatechin	Mayaro virus (MAYV)
SEONG, KIM & SHIN	Wogonin, a flavonoid isolated from Scutellaria baicalensis, has anti-viral activities against	South Korea	2017	Acta Virology	*In vitro*	Wogonin	Influenza A and B

	influenza infection via modulation of AMPK pathways.						
GAUDRY et al	The Flavonoid Isoquercitrin Precludes Initiation of Zika Virus Infection in Human Cells.	France	2018	Int J Mol Sci	*In vitro*	Isoquercitrin (quercetin-3-O-glucoside or Q3G), hyperoside, kaempferol, and quercetin	Zika Virus (ZIKV)
SARWAR et al	Structure activity relationship (SAR) and quantitative structure activity relationship (QSAR) studies showed plant flavonoids as potential inhibitors of dengue NS2B-NS3 protease.	Pakistan	2018	BMC Struct Biol	*In silico*	More than 100 chemical structures of ligand flavonoid molecules	Dengue virus (DENV)
GHOKE et al	Evaluation of antiviral activity of Ocimum sanctum and Acacia arabica leaves extracts against H9N2 virus using embryonated chicken egg model.	India	2018	BMC Complement Altern Med	*In vitro*	Extracts derived from leaves of Acacia arabica.	Influenza H9N2
KERAMAGI & SKARIYACHAN	Prediction of binding potential of natural leads against the prioritized drug targets of chikungunya and dengue viruses by computational screening.	India	2018	3 Biotech	*In silico*	107 compounds from 43 medicinal plants	Chikungunya virus (CHIKV) e Dengue virus (DENV)
HAWAS et al	In vitro inhibition of Hepatitis C virus protease and antioxidant by flavonoid glycosides from the Saudi costal plant Sarcocornia fruticosa.	Egypt	2019	Natural Products Research	*In vitro*	Rhamnazin 3-O-rutinoside (2), rhamnazin 3-O-(6″-O-α-rhamnosyl)-β-galactoside (3), isorhamnetin 3-O-(6″-O-αrhamnosyl)-β-galactoside (4), isorhamnetin 3-O-(2″,6″-O-α-di-rhamnosyl)-β-galactoside (5), and isorhamnetin (6) and allantoin (7); Extracts from leaves of the costal plant Sarcocornia fruticosa.	Hepatitis C virus (HCV)

HÚS et al	Phenanthrenes from Juncus Compressus Jacq. with Promising Antiproliferative and Anti-HSV-2 Activities.	Hungarian	2018	Molecules	*In vitro*	Apigenin and luteolin	Herpes Simplex virus (HSV-2)
KHALIL et al	Guava flavonoid glycosides prevent influenza A virus infection via rescue of P53 activity.	Egypt	2019	J Med Virol	*In silico/In vitro*	Guava extracts and Guava flavonoid glycosides (GFGs) derivatives from leaves extract from green leaves and flowers of guava (Psidium guajava) and lemon (Citrus spp).	Influenza A
GONZÁLES-BÚRGEZ et al.	Comparison between In Vitro Antiviral Effect of Mexican Propolis and Three Commercial Flavonoids against Canine Distemper Virus.	Mexico	2018	Evid Based Complement Alternat Med	*In vitro*	Quercetin, naringenin, and pinocembrin and a flavonoids mixture; Ethanolic Extract of Mexican propolis (EEP).	Canine Distemper Virus (CDV),
MATHEW, AL THANI & YASSINE	Computational screening of known broad-spectrum antiviral small organic molecules for potential influenza HA stem inhibitors.	Qatar	2018	PLoS One	*In silico*	In our in silico docking analysis, 100 natural bioactive flavonoids were initially docked	Influenza A
WOLFF et al	Comprehensive characterisation of polyphenols in leaves and stems of three anti-dengue virus type-2 active Brazilian Faramea species (Rubiaceae) by HPLC-DAD-ESI-MS/MS.	Brazil	2019	Phytochem Anal	*In vitro*	Extracts from species F. bahiensis; The species F. truncata; The species F. hyacinthina; Flavonas e glicosidios, incluindo de kaempferol, quercetin, apigenin, acacetin.	Dengue virus 2 (DENV2)
ZHONG et al	Santin inhibits influenza A virus replication through regulating MAPKs and NF-κB pathways.	China	2019	J Asian Nat Prod Res	*In vitro*	Santin	Influenza A
KIM et al	Cycloartane-type triterpenoid derivatives and a flavonoid glycoside from the burs of Castanea crenata.	South Korea	2019	Phytochemistry	*In vitro*	Kaempferol-3-O-[3″-acetyl-2″,6″-di-E-p-coumaroyl]-β-Dglucopyranoside (16)	Human Rhinovirus (HRV1B), coxsackievirus B3 (CVB3), Influenza A (H1N1) virus

XU et al	Endonuclease Activity Inhibition of the NS1 Protein of Parvovirus B19 as a Novel Target for Antiviral Drug Development.	United States of America	2019	Antimicrob Agents Chemother.	*In vitro*	357 compounds	Human Parvovirus B19V
SADATI et al	Docking study of flavonoid derivatives as potent inhibitors of influenza H1N1 virus neuraminidase.	Iran	2019	Biomed Rep.	*In silico*	Quercetin, catechin, naringenin, luteolin, hispidulin, vitexin, chrysin and kaempferol	Influenza A (H1N1) virus
ÁY et al	Flavonol 7-O-Glucoside Herbacitrin Inhibits HIV-1 Replication through Simultaneous Integrase and Reverse Transcriptase Inhibition.	Hungarian	2019	Evid Based Complement Alternat Med	*In vitro*	Herbacitrin, gossypitrin and quercetin	Human Immunodeficiency Virus (HIV-1)
YIN et al	An antiviral drug screening system for enterovirus 71 based on an improved plaque assay: A potential high-throughput method.	China	2019	J Med Virol	*In vitro*	Nobiletin; kaempferol; morin hydrate; myricetin; taxifolin; baicalin; formononetin; diosmetin; dihydromyricetin	Enterovirus 71 (EV-A71)
LIN et al.	Phloretin inhibits Zika virus infection by interfering with cellular glucose utilisation.	United States of America	2019	Int J Antimicrob Agents	*In vitro*	Phloretin	Zika Virus (ZIKV)
CHEN et al	Assessment of the Effect of Baicalin on Duck Virus Hepatitis.	China	2019	Curr Mol Med	*In vitro/in vivo*	Baicalin	Duck Virus Hepatitis
LEE et al	Antiviral activity of pinocembrin against Zika virus replication.	Singapore	2019	Antiviral Research	*In vitro*	Pinocembrin	Zika Virus (ZIKV)
DAI et al	Antiviral Efficacy of Flavonoids against Enterovirus 71 Infection in Vitro and in Newborn Mice.	China	2019	Viruses	*In vitro/in vivo*	Apigenin, luteolin, diosmetin, tangeretin, nobiletin, galangin, kaempferol, quercetin, myricetin, isorhamnetin, silibinin, liquiritigenin, bavachinin, taxifolin, dihydromyricetin, daidzein, formononetin, epicatechin, chrysosplenetin, and penduletin	Enterovirus 71 (EV-A71)

LEE et al	Antiviral activity of ST081006 against the dengue virus.	Singapore	2019	Antiviral Research	*In vitro*	ST081006, a synthetic flavonoid	Dengue virus (DENV)
JO et al	Characteristics of flavonoids as potent MERS-CoV 3C-like protease inhibitors.	South Korea	2019	Chem Biol Drug Des	*In silico/In vitro*	Herbacetin, isobavachalcone, quercetin 3-β-d-glucoside e helichrysetin	Middle East respiratory syndrome (MERS) Coronaviruses (CoVs)
AKHER et al	Discovery of novel natural flavonoids as potent antiviral candidates against hepatitis C virus NS5B polymerase.	South Africa	2019	Med Hypotheses	*In silico*	43 natural flavonoids	Hepatitis C virus (HCV)
FATEEVA et al	Experimental Study of Flakozid Activity in Viral Hepatitis C In Vitro	Russia	2019	Bulletin of Experimental Biology and Medicine	*In vitro*	Flakosid	Hepatitis C virus (HCV)
LI & WANG	Baicalin inhibits influenza virus A replication via activation of type I IFN signaling by reducing miR-146a.	China	2019	Mol Med Rep	*In vitro/in vivo*	Baicalin	Influenza A
LIU et al	Therapeutic effect of Xanthohumol against highly pathogenic porcine reproductive and respiratory syndrome viruses.	China	2019	Vet Microbiol	*In vitro*	Xanthohumol	Porcine reproductive and respiratory syndrome virus (PRRSV)
GUNASEELAN et al	Prunin suppresses viral IRES activity and is a potential candidate for treating enterovirus A71 infection.	Singapore	2019	Sci Transl Med	*In silico/in vitro*	Prunin	Enterovirus 71 (EV-A71)
BASIC et al	A synthetic derivative of houttuynoid B prevents cell entry of Zika virus.	Germany	2019	Antiviral Research	*In vitro*	Two synthetic houttuynoids TK1023 and TK1024 from the Chinese plant Houttuynia cordata	Zika Virus (ZIKV)
CANTANEO et al	The citrus flavonoid naringenin impairs the in vitro infection of human cells by Zika virus.	Brazil	2019	Scientific Reports	*In silico/In vitro*	Naringenin	Zika Virus (ZIKV)
LOPES et al	Quercetin pentaacetate inhibits in vitro human respiratory syncytial virus adhesion.	Brazil	2020	Virus Research	*In silico/In vitro*	Quercetin (2-(3,4-dihydroxyphenyl)-3,5,7-trihydroxychromen-4-	Orthopneumovirus / Respiratory syncytial virus (RSV)

						one) and Quercetin pentaacetate	
JO et al	Inhibition of SARS-CoV 3CL protease by flavonoids.	South Korea	2020	J Enzyme Inhib Med Chem	*In silico*	Herbacetin, rhoifolin and pectolinarin	Severe respiratory syndrome (SARS-Cov)
DE FREITAS et al	Agathisflavone, a Biflavonoid from Anacardium occidentale L., Inhibits Influenza Virus Neuraminidase.	Brazil	2020	Curr Top Med Chem	*In vitro*	Agathisflavone	Influenza Virus
TSAI et al	Bioactive constituents of Lindernia crustacea and its anti-EBV effect via Rta expression inhibition in the viral lytic cycle.	Taiwan	2020	J Ethnopharmacol	*In vitro*	Apigenin, apigenin-7-O-β-D-glucopyranoside, luteolin-7-O-β-D-glucopyranoside, apigenin-7-O-[β-D-apiofuranosyl (1→6)-β-D-glucopyranoside], apigenin-7-O-[α-L-rhamnopyranosyl (1→2)-β-D-glucopyranoside]	Epstein-Barr Virus (EBV)
LING et al	Flavonoids from Houttuynia cordata attenuate H1N1-induced acute lung injury in mice via inhibition of influenza virus and Toll-like receptor signalling.	China	2020	Phytomedicine	*In vitro/in vivo*	Rutin, hyperin, isoquercitrin and quercitrin; Extract from Houttuynia cordata Thunb. (Saururaceae).	Influenza A (H1N1) virus
CARE et al	Discordant Activity of Kaempferol Towards Dengue Virus and Japanese Encephalitis Virus.	Thailand	2020	Molecules	*In vitro*	Kaempferol	Japanese encephalitis virus (JEV) e Dengue Vírus (DENV)
LI et al	Inhibition of herpes simplex virus by myricetin through targeting viral gD protein and cellular EGFR/PI3K/Akt pathway.	China	2020	Antiviral Research	*In vitro/in vivo/in silico*	Myricetin	Herpes Simplex Vírus (HSV-2)
ZOU et al	Structure-activity relationship of flavonoid bifunctional inhibitors against zika virus infection.	China	2020	Biochem Pharmacol	*In vitro*	Galangin, kaempferide, myricetin, quercetin, dihydromyricetin,	Zika Virus (ZIKV)

						trifloroside and epigallocatechin gallate (EGCG)	
YU et al	Computational screening of antagonists against the SARS-CoV-2 (COVID-19) coronavirus by molecular docking	China	2020	Int J Antimicrob Agents	*In silico*	Luteolin	Severe respiratory syndrome 2 (SARS-Cov-2)
NGWA et al	Potential of Flavonoid-Inspired Phytomedicines against COVID-19	United States of America	2020	Molecules	*In silico/In vitro*	Hesperetin, Myricetin, Linebacker, Caflanone, Equivir	Severe respiratory syndrome 2 (SARS-Cov-2)
TIAN et al	Dihydromyricetin is a new inhibitor of influenza polymerase PB2subunit and influenza-induced inflammation	China	2020	Microbes and Infection	*In silico/In vitro*	Dihydromyricetin	Influenza A (H1N1) virus
HAMZA et al	nCOV-19 peptides mass fingerprinting identification, binding, and blocking of inhibitors flavonoids and anthraquinone of Moringa oleifera and hydroxychloroquine	Pakistan	2020	J biomol struct dyn	*In silico*	Kaempferol	Severe respiratory syndrome 2 (SARS-Cov-2)
KHANDELWAL et al	Antiviral activity of Apigenin against buffalopox: Novel mechanistic insights and drug-resistance considerations	India	2020	Antiviral research	*In vitro/In vivo*	Apigenin	Buffalopox virus (BPXV)
CHU et al	Wogonin inhibits in vitro herpes simplex virus type 1 and 2 infection by modulating cellular NF-κB and MAPK pathways	China	2020	BMC Microbiol	*In vitro*	Wogonin	Herpes simplex virus 1 and 2 (HSV-1 and HSV-2)
JO et al	Flavonoids with inhibitory activity against SARS-CoV-2 3CLpro	South Korea	2020	J Enzyme Inhib Med Chem	*In silico/In vitro*	70 flavonoids	Severe respiratory syndrome 2 (SARS-Cov-2)
XU et al	Apigenin suppresses influenza A virus-induced RIG-I activation and viral replication	China	2020	J Med Virol	*In vitro*	Apigenin	Influenza A (H1N1) virus
HONG et al	Morin Hydrate Inhibits Influenza Virus entry into Host Cells and Has Anti-inflammatory Effect in Influenza-infected Mice	South Korea	2020	Immune Netw.	*In vitro/In vivo*	Morin hydrate	Influenza A (H1N1) virus
MENDES et al	The anti-Zika virus and anti-tumoral activity of the citrus flavanone lipophilic naringenin-based compounds	Brazil	2020	Chem Biol Interact	*In vitro*	Narigenin derivatives	Zika Virus (ZIKV)

BOONYASUPPAYAKORN et al	Dibromopinocembrin and Dibromopinostrobin Are Potential Anti-Dengue Leads with Mild Animal Toxicity	Thailand	2020	Molecules	*In vitro/In vivo*	Modified flavanones, pinocembrin and pinostrobin	Dengue Virus 2 (DENV2)
KWON et al	Protective Effect of Flavonoids from Ohwia caudata against Influenza a Virus Infection	South Korea	2020	Molecules	*In vitro*	2'-hydroxyl yokovanol, 2'-hydroxyl neophellamuretin, yokovanol, swertisin, spinosin, and 7-methyl-apigenin-6-C-β-glucopyranosyl 2″-O-β-d-xylopyranoside	Influenza A (H1N1) virus
LI et al	Inhibitory Activity of Honeysuckle Extracts against Influenza A Virus In Vitro and In Vivo	China	2020	Virol. Sin.	*In vitro/In vivo*	Honeysuckle flavonoid-rich fraction	Influenza A (H1N1, H3N2 and H1N1-H275Y) virus
BASU, SARKAR and MAULIK	Molecular docking study of potential phytochemicals and their effects on the complex of SARS-CoV2 spike protein and human ACE2	India	2020	Sci Rep	*In silico*	Hesperidin and Chrysin	Severe respiratory syndrome 2 (SARS-Cov-2)
MANDOUR, ZLOTOS and SALEM	A multi-stage virtual screening of FDA-approved drugs reveals potential inhibitors of SARS-CoV-2 main protease	Egypt	2020	J biomol struct dyn	*In silico*	Rutin	Severe respiratory syndrome 2 (SARS-Cov-2)
RAMESHKUMAR et al	Computational selection of flavonoid compounds as inhibitors against SARS-CoV-2 main protease, RNA-dependent RNA polymerase and spike proteins: A molecular docking study	Saudi Arabia	2020	Saudi J Biol Sci.	*In silico*	458 flavonoids	Severe respiratory syndrome 2 (SARS-Cov-2)
ZHOU et al	Anti-HSV-1 effect of dihydromyricetin from Ampelopsis grossedentata via the TLR9-dependent anti-inflammatory pathway	China	2020	J Glob Antimicrob Resist	*In vitro*	Dihydromyricetin	Herpes simplex virus 1 (HSV-1)
GOGOI et al	Computational guided identification of a citrus flavonoid as potential inhibitor of SARS-CoV-2 main protease	India	2020	Mol Divers	*In silico*	44 citrus flavonoids	Severe respiratory syndrome 2 (SARS-Cov-2)

XIONG et al	Isolation and identification of two new compounds from the seeds of Moringa oleifera and their antiviral and anti-inflammatory activities	China	2020	Nat Prod Res	*In vitro*	Vitexin	Influenza A (H1N1) virus
VERMA, HENDERSON and SHEN et al	Proton-Coupled Conformational Activation of SARS Coronavirus Main Proteases and Opportunity for Designing Small-Molecule Broad-Spectrum Targeted Covalent Inhibitors	United States of America	2020	J Am Chem Soc.	*In silico*	Galangin	Severe respiratory syndrome 2 (SARS-Cov-2)
KUMAR et al	In silico studies reveal antiviral effects of traditional Indian spices on COVID-19	India	2020	Curr Pharm Des	*In silico*	Myricetin, Rutin, Luteolin, Scopolin, Apigenin, others	Severe respiratory syndrome 2 (SARS-Cov-2)
JAIN et al	In silico evaluation of flavonoids as effective antiviral agents on the spike glycoprotein of SARS-CoV-2	India	2020	Saudi J Biol Sci.	*In silico*	Rutin, quercetin, naringin, morin, luteolin, hesperetin, galangin, fisetin, chrysin, apigenin	Severe respiratory syndrome 2 (SARS-Cov-2)
Hamza et al	nCOV-19 peptides mass fingerprinting identification, binding, and blocking of inhibitors flavonoids and anthraquinone of Moringa oleifera and hydroxychloroquine	Pakistan	2021	J Biomol Struct Dyn.	*In silico/In vitro*	*Moringa oleifera* flavonoid extract	Severe respiratory syndrome 2 (SARS-Cov-2)
Li et al	Inhibitory Activity of Honeysuckle Extracts against Influenza A Virus In Vitro and In Vivo	China	2021	Virol Sin.	*In vitro/In vivo*	Honeysuckle flavonoid extract	Influenza A H1N1, H3N2 and H1N1-H275Y
Dubey and Dubey	Molecular Docking Studies of Bioactive Nicotiflorin against 6W63 Novel Coronavirus 2019 (COVID-19)	India	2021	Comb chem High Throughput Screen.	*In silico*	Nicotiflorin	Severe respiratory syndrome 2 (SARS-Cov-2)
Rameshkumar et al	Computational selection of flavonoid compounds as inhibitors against SARS-CoV-2 main protease, RNA-dependent RNA polymerase and spike proteins: A molecular docking study	Saudi Arabia	2021	Saudi J Biol Sci.	*In silico*	458 flavonoid compounds	Severe respiratory syndrome 2 (SARS-Cov-2)
Gogoi et al	Computational guided identification of a citrus flavonoid as potential inhibitor of SARS-CoV-2 main protease	India	2021	Mol Divers.	*In silico*	44 citrus flavonoids	Severe respiratory syndrome 2 (SARS-Cov-2)

Kumar et al	In Silico Studies Reveal Antiviral Effects of Traditional Indian Spices on COVID-19	India	2021	Curr Pharm Des.	*In silico*	75 compounds, among them myricetin, isovitexin, rutin, astragalin, luteolin, and apigenin	Severe respiratory syndrome 2 (SARS-Cov-2)
Jain et al	In silico evaluation of flavonoids as effective antiviral agents on the spike glycoprotein of SARS-CoV-2	India	2021	Saudi J biol Sci.	*In silico*	Apigenin, chrysin, fisetin, galangin, hesperetin, luteolin, morin, naringin, quercetin and rutin	Severe respiratory syndrome 2 (SARS-Cov-2)
Leal et al	Amazonian *Siparuna* extracts as potential anti-influenza agents: Metabolic fingerprinting	Brazil	2021	J Ethnopharmacol.	*In vitro*	Flavonoid rich fraction	Influenza A (H1N1) virus
Potshangbam et al	Phenylbenzopyrone of Flavonoids as a Potential Scaffold to Prevent SARSCoV-2 Replication by Inhibiting its M PRO Main Protease	India	2021	Curr Pharm biotechnol.	*In silico*	Screening of natural compound libraries	Severe respiratory syndrome 2 (SARS-Cov-2)
Bhowmmik et al	In silico validation of potent phytochemical orientin as inhibitor of SARS-CoV-2 spike and host cell receptor GRP78 binding	India	2021	Heliyon.	*In silico*	Orientin	Severe respiratory syndrome 2 (SARS-Cov-2)
D'Angeli et al	Antimicrobial, Antioxidant, and Cytotoxic Activities of Juglans regia L. Pellicle Extract	Italy	2021	Antibiotics	*In silico*	*Juglans regia* L. Pellicle Extract	Herpes simplex virus 1 and 2 (HSV-1 and HSV-2)
Schonhofer et al	Flavonoid-based inhibition of cyclin-dependent kinase 9 without concomitant inhibition of histone deacetylases durably reinforces HIV latency	Canada	2021	Biochem Pharmacol.	*In vitro*	chrysin, apigenin, luteolin, flavopiridol, luteolin and luteolin-7-glucoside	Human immunodeficiency virus (HIV)
Swain et al	Anti-HIV-drug and phyto-flavonoid combination against SARS-CoV-2: a molecular docking-simulation base assessment	India	2021	J Biomol Struct Dyn.	*In silico*	apigenin, catechin, di-hydroquercetin, epi-gallocatechin gallate, hesperidin, LPRP-Et-97543, quercetin, quercetin-3-rhamnoside and rutin	Severe respiratory syndrome 2 (SARS-Cov-2)

Lima et al	Flavonoids from Pterogyne nitens as Zika virus NS2B-NS3 protease inhibitors	Brazil	2021	Bioorg Chem.	*In silico*	Screening of 150 natural compounds, including quercetin, rutin and pedalitin	Zika Virus (ZIKV)
Rakshit et al	Flavonoids as potential therapeutics against novel coronavirus disease-2019 (nCOVID-19)	India	2021	J Biomol Struct Dyn.	*In silico*	Rhoifolin, 5,7dimethoxyflavanone-40-O-b-glucopyranoside, baicalin, luteolin, kaempferol, isoquercetin, tamarixetin, 5-Hydroxy-3,4,7-trimethoxy-flavonone , euparotine, diosmetin, daidzein, nepitrin, taxifolin, hesperidin	Severe respiratory syndrome 2 (SARS-Cov-2)
Nair et al	Artemisia annua L. extracts inhibit the in vitro replication of SARS-CoV-2 and two of its variants	United States of America	2021	J Ethnopharmacol.	*In vitro*	Flavonoid-rich extract	Severe respiratory syndrome 2 (SARS-Cov-2)
Kumari and Subbarao	Deep learning model for virtual screening of novel 3C-like protease enzyme inhibitors against SARS coronavirus diseases	India	2021	Comput Biol Med.	*In silico*	327 flavonoids	Severe respiratory syndrome 2 (SARS-Cov-2)
Rahman et al	Molecular docking analysis of rutin reveals possible inhibition of SARS-CoV-2 vital proteins	India	2021	J tradit complement Med.	*In silico*	Rutin	Severe respiratory syndrome 2 (SARS-Cov-2)
Bodoruske et al	Wild Sambucus nigra L. from north-east edge of the species range: A valuable germplasm with inhibitory capacity against SARS-CoV2 S-protein RBD and hACE2 binding in vitro	Latvia	2021	Ind Crops Prod	*In vitro*	Flavonoid-rich extract	Severe respiratory syndrome 2 (SARS-Cov-2)
Zhang et al	Flavonoid-triazolyl hybrids as potential anti-hepatitis C virus agents: Synthesis and biological evaluation	China	2021	Eur J Med Chem.	*In vitro*	19 synthetic flavonoid derivatives	Hepatitis C virus (HCV)

Bhati et al	Rational design of flavonoid based potential inhibitors targeting SARS-CoV 3CL protease for the treatment of COVID-19	India	2021	J Mol. Struct.	*In silico*	15 flavonoid-based compounds and quercetin	Severe respiratory syndrome 2 (SARS-Cov-2)
Zhan et al	Potential antiviral activity of isorhamnetin against SARS-CoV-2 spike pseudotyped virus in vitro	China	2021	Drug Dev Res.	*In vitro*	Flavonoid-rich fraction, quercetin and isorhamnetin	Severe respiratory syndrome 2 (SARS-Cov-2)
Zandi et al	Baicalein and Baicalin Inhibit SARS-CoV-2 RNA-Dependent-RNA Polymerase	United States of America	2021	Microorganisms	*In vitro*	Baicalein and baicalin	Severe respiratory syndrome 2 (SARS-Cov-2)
Mishra et al	The interaction of the bioflavonoids with five SARS-CoV-2 proteins targets: An in silico study	United States of America	2021	Comput Biol Med.	*In silico*	85 flavonoid compounds	Severe respiratory syndrome 2 (SARS-Cov-2)
Su et al	Identification of pyrogallol as a warhead in design of covalent inhibitors for the SARS-CoV-2 3CL protease	China	2021	Nat Commun.	*In silico/In vitro*	Myricitin and derivatives	Severe respiratory syndrome 2 (SARS-Cov-2)
Rudrapal et al	In silico screening of phytopolyphenolics for the identification of bioactive compounds as novel protease inhibitors effective against SARS-CoV-2	India	2021	J Biol Dtruct Dyn.	*In silico*	Taxifolin, eriodictyol, leucopelargonidin, morin and myricetin	Severe respiratory syndrome 2 (SARS-Cov-2)
Mangiavacchi et al	Seleno-Functionalization of Quercetin Improves the Non-Covalent Inhibition of Mpro and Its Antiviral Activity in Cells against SARS-CoV-2	Italy	2021	Int J Mol Sci.	*In silico/In vitro*	Selenium and tellurium-quercetin derivatives	Severe respiratory syndrome 2 (SARS-Cov-2)
Jiménez-Avalos et al	Comprehensive virtual screening of 4.8 k flavonoids reveals novel insights into allosteric inhibition of SARS-CoV-2 M PRO	Peru	2021	Sci Rep.	*In silico*	4800 flavonoids	Severe respiratory syndrome 2 (SARS-Cov-2)
Xiong et al	Flavonoids in Ampelopsis grossedentata as covalent inhibitors of SARS-CoV-2 3CL pro: Inhibition potentials, covalent binding sites and inhibitory mechanisms	China	2021	Int J Biol Macromol.	*In silico/In vitro*	Ampelopsis grossedentata flavonoids, including dihydromyricetin, isodihydromyricetin and myricetin	Severe respiratory syndrome 2 (SARS-Cov-2)

Kim et al	Antiviral Activity of Chrysin against Influenza Virus Replication via Inhibition of Autophagy	China	2021	Viruses	*In vitro*	Chrysin	Influenza A (H1N1) virus
Umar	Flavonoid compounds of buah merah (Pandanus conoideus Lamk) as a potent SARS-CoV-2 main protease inhibitor: in silico approach	Indonesia	2021	Futur J Pharm Sci.	*In silico*	Quercetin 3'-glucoside, quercetin 3-O-glucose, and taxifolin 3-O-α-arabinopyranos	Severe respiratory syndrome 2 (SARS-Cov-2)
Elhusseiny et al	Antiviral, Cytotoxic, and Antioxidant Activities of Three Edible Agaricomycetes Mushrooms: Pleurotus columbinus, Pleurotus sajor-caju, and Agaricus bisporus	Egypt	2021	J Fungi	*In vitro*	Flavonoid-rich extract	Adenovirus type 7 (Ad7) and Herpes simplex virus 2 (HSV-2)
Rehman et al.	Effectiveness of Natural Antioxidants against SARS-CoV-2? Insights from the In-Silico World	Pakistan	2021	Antibiotics	*In silico*	Various flavonoids	Severe respiratory syndrome 2 (SARS-Cov-2)
Lalani, Masomian and Poh	Functional Insights into Silymarin as an Antiviral Agent against Enterovirus A71 (EV-A71)	Malasya	2021	In J Mol Sci.	*In silico/In vitro*	Silymarin	Enterovirus A71
Chiou et al	Ugonin J Acts as a SARS-CoV-2 3C-like Protease Inhibitor and Exhibits Anti-inflammatory Properties	Taiwan	2021	Front Pharmacol.	*In silico/In vitro*	Ugonin J	Severe respiratory syndrome 2 (SARS-Cov-2)
Panagiotopoulos et al	Natural Polyphenols Inhibit the Dimerization of the SARS-CoV-2 Main Protease: The Case of Fortunellin and Its Structural Analogs	Greece	2021	Molecules	*In vitro*	Fortunellin	Severe respiratory syndrome 2 (SARS-Cov-2)
Alomair et al	In Silico Prediction of the Phosphorylation of NS3 as an Essential Mechanism for Dengue Virus Replication and the Antiviral Activity of Quercetin	United States of America	2021	Biology	*In silico*	Quercetin	Dengue Virus (DENV)
Long, Zhao and Wu	Hesperetin inhibits KSHV reactivation and is reversed by HIF1α overexpression	China	2021	J Gen Virol	*In vitro*	Hesperetin	Kaposi's sarcoma-associated herpesvirus (KSHV)

Al-Karmalawy et al	Naturally Available Flavonoid Aglycones as Potential Antiviral Drug Candidates against SARS-CoV-2	Egypt	2021	Molecules	*In silico/In vitro*	taxifolin, pectolinarigenin, tangeretin, gardenin B, and hispidulin	Severe respiratory syndrome 2 (SARS-Cov-2)
Xiao et al	Both Baicalein and Gallocatechin Gallate Effectively Inhibit SARS-CoV-2 Replication by Targeting Mpro and Sepsis in Mice	China	2021	Inflammation	*In silico/In vitro*	35 flavonoids	Severe respiratory syndrome 2 (SARS-Cov-2)
Hengphasatporn et al	Alkyne-Tagged Apigenin, a Chemical Tool to Navigate Potential Targets of Flavonoid Anti-Dengue Leads	Thailand	2021	Molecules	*In silico/In vitro*	Alkyne-tagged apigenin	Dengue Virus 2 (DENV2)
Jantakee et al	Anti-Herpes Simplex Virus Efficacy of Silk Cocoon, Silkworm Pupa and Non-Sericin Extracts	Thailand	2021	Antibiotics	*In vitro*	Flavonoid-rich extract	Herpes simplex virus 1 and 2 (HSV-1 and HSV-2)
Melk et al	Antiviral Activity of Zinc Oxide Nanoparticles Mediated by Plumbago indica L. Extract Against Herpes Simplex Virus Type 1 (HSV-1)	Egypt	2021	Int J Nanomedicine	*In vitro*	Flavonoid-rich extract	Herpes simplex virus 1 (HSV-1)
Attallah	Promising Antiviral Activity of Agrimonia pilosa Phytochemicals against Severe Acute Respiratory Syndrome Coronavirus 2 Supported with In Vivo Mice Study	Egypt	2021	Pharmaceuticals	*In vivo*	Flavonoid-rich extract	Severe respiratory syndrome 2 (SARS-Cov-2)
